# Money counts: effects of monetary vs. purely numerical values on the mental representation of quantities

**DOI:** 10.1007/s00426-025-02118-z

**Published:** 2025-04-11

**Authors:** Gianluca Grimalda, Giovanni Ottoboni, Alessandro Cappellini, Mario Bonato, Mariagrazia Ranzini

**Affiliations:** 1 Passau University, Passau, Germany; 2 Department of Psychology, University of Bologna, Viale Berti Pichat 5, Bologna, 40127 Italy; 3 Intesa Sanpaolo S.p.A., Torino, Italy; 4 United International Business School (UIBS), Geneva, Switzerland; 5 Department of General Psychology (DPG), University of Padova, Padua, Italy

**Keywords:** Quantity processing, Quantity representation, Number processing, Money processing, Utility theory

## Abstract

It has been established that humans use different cognitive models to represent and process numerical quantities. In this study, we investigated whether the representation of monetary values fundamentally differs from the representation of numbers. We also examined the influence of both socio/economic factors and mathematical ability on such representation. A group of adults (N = 272) were tested anonymously with a variant of the number-to-position task (Siegler & Opfer, 2003). They were asked to position on a horizontal line quantities expressed either in numerical format (e.g., 50) in the “Number” conditions or as monetary values (e.g., 50€) in the “Money” conditions. The extremes of the line consisted either of specific values (i.e. “2 or 2€” and “503 or 503€”) in the “Fixed” conditions or of unspecific concepts of quantity (e.g., “little” and ”a lot”) in the “Fuzzy” conditions. A linear model, as opposed to a logarithmic one, provided the best fit of group average data in all experimental conditions except for the “Money-Fuzzy” condition. The percentages of absolute error were significantly larger for Money stimuli than Number stimuli in both Fixed and Fuzzy conditions. This is consistent with the law of diminishing marginal utility, which entails that the value of monetary quantities is described by a concave curve rather than a linear relationship. As expected from previous research, participants who were more used to spending large quantities of money were closer to the linear representation model. Participants with higher mathematical abilities represented numerical values more closely to a linear model, but no such effect was found for monetary quantities.

**Supplementary Information:**

The online version contains supplementary material available at 10.1007/s00426-025-02118-z.

## Introduction

Research on numerical cognition has revealed several characteristics of the way people process and represent numerical quantities (Dehaene & Cohen, 1995; Hubbard, Piazza, Pinel, & Dehaene, 2005). Cognitive and cultural factors contribute to modelling the interplay between the improvement of one’s general cognitive abilities during development and the culture-based increased familiarity with different magnitudes after repeated exposure (Ansari, 2008). Most studies investigating numerical representation considered numbers independently from the context in which they were adopted. However, while numbers are intrinsically abstract entities, they are often associated with specific metric units in their everyday use. One of the most important of such scales is money. The two key questions tackled in this study are whether individuals use different mental models to represent abstract numerical quantities vis-à-vis money quantities and which role socioeconomic or cognitive factors play in characterizing such representations.

Some previous studies investigated the impact of the introduction of the Euro on the cognitive representation of monetary quantities. Dehaene and Marques (2002) tested students from European universities in the period immediately preceding the introduction of the Euro currency. They observed increased variability in price estimation as a function of the real price of the item to be estimated. This variability was well captured by a logarithmic-scaled function, and it was moderated by purchase frequency and estimated price variability. Importantly, when comparing estimation performance in familiar (current currency) and unfamiliar (Euros) currencies, a less precise quantity representation was observed with the unfamiliar currency. Marques and Dehaene (2004) later showed that price estimations became progressively more accurate after the introduction of the Euro. This increased accuracy was a function of purchase frequency.

Convergent evidence from psychological and marketing studies indicates that individuals’ perception of economic values is driven by multiple psychological factors. In particular, it has been suggested that the subjective way consumers perceive prices (i.e., a price can be subjectively judged as high or low for a specific product) and the difference between them (i.e., a price can be subjectively judged as higher or lower for a specific product compared to the cost of another product), reflects mental representations which are similar to the mental representation of abstract numerical quantities. Indeed, similar to numerical estimations, different types of cues have been shown to play a role in the evaluation economic values, cues such as the spatial location of products (e.g., Cai et al., 2012; Giuliani et al., 2017; Valenzuela & Raghubir, 2013), the congruency between numerical and physical information in comparison of coins (e.g., Fitousi, 2010; Goldman et al., 2012), the difference between the leftmost digits in comparative judgments (Thomas & Morwitz, 2005), or the association between value and response side (Spatial Numerical Association of Response Codes: SNARC effect; Dehaene et al., 1993) recently found with economic values (Giuliani et al., 2024). Taken together, these findings indicate that several effects observed in price-related judgements are similar to those observed with abstract numbers and can be explained by cognitive models of numerical cognition (Thomas & Morwitz, 2005; see also Giuliani et al., 2017; Fitousi, 2010; Goldman et al., 2012).

To map the mental representation of quantities, we used a variant of the number-to-position task (NPT) devised initially by Siegler and Opfer (2003) to compare the performance of primary school children and adults in positioning an Arabic number on a horizontal line. In the original NPT task, two ranges were used, one from 0 to 100 and another from 0 to 1000. The extremes of the line indicated the range of numbers to represent (0 vs. 100 or 0 vs. 1000). The performance of second and fourth graders with the range 0-100 was better approximated by a linear model than a logarithmic model, whereas the opposite was true for the range 0-1000. However, sixth graders and adults’ performance were predominantly linear for both ranges, indicating increased precision in the numerical representation as a function of increasing numerical knowledge (Siegler, 2016). The original findings by Siegler and Opfer (2003) were replicated and extended in several other studies (e.g., Berteletti, Lucangeli, Piazza, Dehaene, & Zorzi et al., 2010; Geary, 2011; Booth & Siegler, 2006; Sella, Sader, Lolliot, & Cohen Kadosh, 2016; Siegler & Booth, 2004; Sasanguie et al., 2012). Importantly, these additional studies have shown that linearity in the NPT is correlated with both mathematical achievement, such as in 6 to 8 years-old children (Booth & Siegler, 2006), and with acquaintance with digits and magnitude ordering in preschoolers (e.g., Berteletti et al., 2010). This suggests that experience with number concepts improves children’s ability to represent the distance between numerical magnitudes. This may be explained by a progressively increased familiarity with algebraic computations and with the development of better mathematical skills (see also: Booth & Siegler, 2008; Geary, 2011; LeFevre et al., 2013; Sasanguie et al., 2012; Sasanguie et al., 2013; Sella, Berteletti, Brazzolotto, Lucangeli, & Zorzi, 2013). The strong association between number line estimation and mathematical skills has been confirmed by a meta-analysis of more than forty studies (Schneider et al., 2018).

Formal education plays a crucial role in developing an accurate mental representation of magnitudes, as proven in a study involving the Mundurucu, an Amazonian population with a very limited lexicon for numbers (i.e., one, two, more than two) and restricted access to formal education (Dehaene et al., 2008). Both uneducated and educated Mundurucu participants tended to place Mundurucu numerals logarithmically in space. However, when educated, Mundurucu were presented with numbers as spoken numerals (Portuguese), an effect of formal education emerged. Participants with few years of education still placed numbers logarithmically, while those with a longer education span placed numbers linearly. This finding suggests that the linear number line might be a cultural construct that does not develop without formal education, while the core innate and universally shared mapping could be logarithmic (Dehaene et al., 2008).

Logarithmic vs. linear mappings have been shown with various tasks and interpreted as directly representing changes in the distance between numbers in an internal mental number line (Dehaene et al., 2008), even if alternative interpretations and analyses have been advanced. In particular, it has been argued that participants tend to implement strategies to solve the NTP task (e.g., proportional reasoning), which does not necessarily imply the existence of a linear/logarithmic internal mapping (e.g., Barth & Paladino, 2011; Slusser et al., 2013). Specifically, many studies have observed that strategies based on a mental division of the line and on the use of points on the line as anchors (e.g., the midpoint) develop with increasing familiarity with numbers during childhood and are used in adulthood (e.g., Peeters et al., 2016, 2017; Rouder & Geary, 2014; Sella et al., 2016; Sullivan et al., 2011; White & Szűcs, 2012). Studies advancing different accounts found useful the use of cyclical power models to investigate the performance at the NPT task. Interestingly, the number of cycles in the model varies in function of the familiarity that children have with numbers (Ebersbach et al., 2008, 2015, 2016; Moeller et al., 2009).

**Fig. 1:**
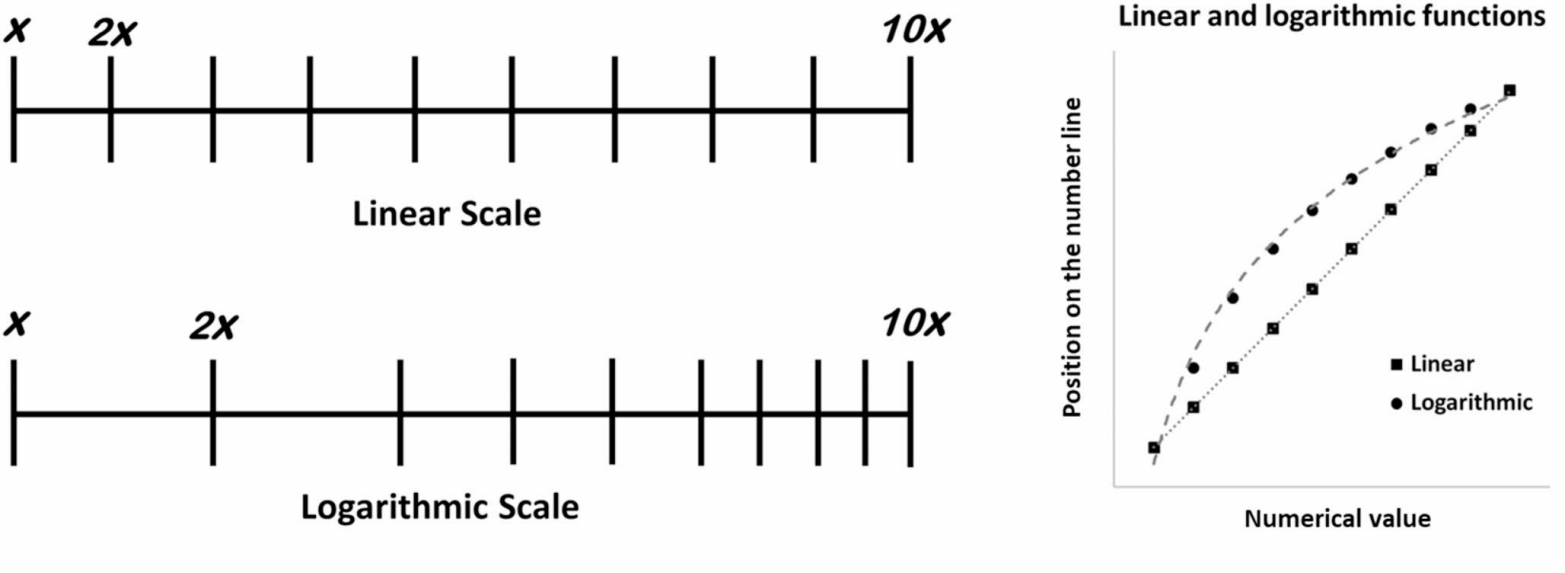
A schematic visual representation of linear or logarithmic number line scaling is depicted on the left. On the right, linear and logarithmic estimates of the numerical values (i.e. positions on the number line) are plotted: in the logarithmic scaling, the increasing values fall progressively closer one to the other; differently, they are equidistant in the linear scaling

Beyond the participants’ strategy, the logarithmic function captures the overestimation of smaller numbers and the progressive placement of larger numbers closer to each other. In contrast, higher degrees of accuracy (in terms of accurate distance between magnitudes) are well captured by the linear function, not only in children but also in adult participants, and linearity is usually considered a good indicator of accurate performance (e.g., Sella 2016). Linear and logarithmic numerical mappings are schematically illustrated in Fig. 1. Considering that the focus of the current study is to investigate performance in the NTP task in association with numerical values and amount of money, we used logarithmic and linear models as methods of analysis. Moreover, evidence indicates that PAE, log-linear model, one-cycle cyclical power model, and two-cycle cyclical power model are correlated and are similarly predicted by the same factors (Gross et al., 2018). Finally, although the idea that logarithmic and linear fits mirror internal mental representations is debated, it is important to highlight that the idea of a logarithmic representation of monetary values reflects hypotheses advanced in economics (e.g., marginal utility). Therefore, a methodology based on a comparison between linear and logarithmic representations seems adequate and useful for testing skills related to the processing of numerical quantities concerning potential interacting factors (e.g., age, level of education, quantity range). Importantly, the NPT approach has also been used to investigate the mental representation of money quantities. A study by Iuculano and Butterworth (2011) contrasted the performance of adults and children aged 10 while positioning numerical quantities in different formats. The different formats included integers (0-100), fractions or decimals (0–1), and money values (0p-1£). They found remarkably similar, if not identical, patterns across groups and formats except for fractions, which were processed slowly and with more errors (see also Bonato et al., 2007). In the present study, we investigated the characteristics of the representation of quantities of money compared to that of numbers. We also explored the influence of either socio-economic or cognitive factors on the number and money representations. Participants were required to position on a line quantities expressed either in numerical format (e.g., the Arabic number 50) or as monetary values (e.g., the image of a banknote of 50€).

We also manipulated the values visualized at the extremes of the line. Line bounds could be either precise values (“2” and “503”, or “2€” and “503€”) or fuzzy values (“small” and “large”, or “little” and ”much”). We adopted the fuzzy range of bounds because we were interested in investigating the differences between money and numbers when bounds may prompt individuals to use a subjective scale rather than an objective one. To our knowledge, this manipulation has never been tested in the domain of numerical cognition, while it resembles paradigms of subjective price estimations used for consumer studies (Thomas & Morwitz, 2005). Given our different formats of stimuli compared with Siegler and Opfer (2003), we prefer to call our task “Quantity to Position“ task (QPT) rather than NPT. Furthermore, we collected information on participants’ socio-economic status and spending habits, and we developed a series of questions to evaluate mathematical abilities related to the use of basic computational principles.

We predicted both similarities and differences between numerical and monetary stimuli. Concerning similarities, since numbers are abstract concepts, we expected that the way they are represented similarly applies to different contexts. Therefore, we predicted that (*H*_*1*_) individuals’ performance in the QPT would be better captured by a linear function both for numerical and monetary stimuli in the *Fixed* condition, which is in line with previous studies. Indeed, in their NTP task, Iuculano and Butterworth (2011) reported similar performance when adults and children processed several quantities formats, including numbers and money. They found that adults and 10-year-old children placed linearly a small amount of number and money quantities presented in numerical format. However, the existing evidence shows that adults still rely on a logarithmic representation with different tasks, for instance, when presented with non-symbolic entities (e.g., sets of dots: Dehaene et al., 2008; Izard & Dehaene, 2007; Piazza et al., 2004). Another aspect that appears to be relevant is that manipulating line bounds affects NPT responses (Rips, 2012; Cohen & Blanc-Goldhammer, 2011). We argue that this should particularly be the case when the labels we used to define the extremes in our QPT are liable to subjective interpretation (i.e., “Small-“Large’, “Little”-“Much”). Therefore, we expect that (*H*_*2*_) fuzzy bounds will significantly affect the representation of quantities, either numbers or money and that individuals’ percentage of absolute error (PAE), corresponding to the deviation from the linear representation, will be larger in the *Fuzzy* condition than in the *Fixed* condition.

As for the differences between number and monetary quantities, we conjecture that (*H*_*3*_) higher PAE will be found in the Money conditions compared to the Number condition. This hypothesis is grounded in a long-standing tradition in decision theory that posits the *principle of decreasing marginal utility*. In the words of Berkman et al. (2016), “The principle refers to the phenomenon that each additional unit of consumption leads to an ever-smaller increase in subjective value. For example, three bites of candy are better than two bites, but the twentieth bite does not add much to the experience beyond the nineteenth (and could even make it worse).” The well-known “water and diamond” paradox originally proposed by Adam Smith may be seen as another instance of the principle of decreasing marginal utility. The paradox can be rephrased as follows: A drop of water is immensely valuable to a person dying of thirst in the desert, while a drop of water is worth nothing to a person who has water cheaply supplied to their tap. In microeconomics, individuals derive utility from the consumption of goods and services. Since money is often used to consume such goods and services, we can think of utility as defined by money itself.^1^ A direct consequence of the principle of decreasing marginal utility is that the representation of the so-called utility function - namely, the function mapping amounts of money into the *value* a person derives from such money – has a concave shape. The principle of decreasing marginal utility was originally proposed by Bernoulli (1738) to solve a paradox involving decisions under risk. Incidentally, Bernoulli (1738) proposed a logarithmic function to model individual utility. The principle of decreasing marginal utility was later incorporated into mainstream economics to model individual consumption choices. It featured prominently in the works of both Keynes (1937) and von Neumann and Morgenstern (1947). Pratt (1964) and Arrow (1971) provided a general axiomatic approach in which a risk aversion parameter determines the concavity of the utility function. Under this approach, the logarithmic case is special among the family of concave utility functions. Although the basic model has been considerably enriched (e.g., Kahnemann & Tverski, 1979) and is still subject to extensive research (e.g., Carroll & Kimball, 1996; Clark & Oswald, 1998; Rabin, 2000), decreasing marginal utility and the concavity of the representation of money is a widely-held principle in economics. A straightforward assumption is that when individuals are asked to represent money in our QPT, they will immediately represent the value in terms of the purchasing capability associated with a certain quantity of money. Consequently, the decreasing marginal utility principle predicts that the “value” (or utility) that individuals associate with progressively larger quantities should decrease more than the increase in the quantity itself. Decreasing marginal utility entails both larger deviations from the linear model and higher frequency of the logarithmic model in the Money than the Number conditions.

Finally, we also predict that socioeconomic, educational/cultural, or cognitive aspects of an individual’s life contribute to shaping quantities representations. There is ample evidence that exposure and training with quantities increase familiarity, thus increasing the precision in representing and manipulating them (e.g., Ansari, 2008). We, therefore, hypothesized (*H*_*4*_) that people who were more accustomed to dealing with large quantities of money in real life would also be more likely to come closer to a linear representation: the higher the quantity of individuals’ spending habits, the closer their performance in the QPT to the linear model. This should be truer in the Money conditions than in the Number conditions. This prediction builds on the idea that spending habits significantly affect individuals’ numeric and money representation (Ungemach et al., 2011; Vlaev et al., 2011). However, the same could be true for those occupied in so-called STEM professions, which require computational skills and, more generally, in high-income occupations. We symmetrically hypothesized (*H*_*5*_) that the higher the individuals’ ability in Mathematics, the closer their performance in the QPT to the linear model. This should be particularly true in the Number than in the Money conditions. We construct two scales in our post-experiment questionnaire to probe into these hypotheses. One taps into familiarity with monetary transactions in real life, and another one with ability in maths and probability. In our regressions, we also control for a large vector of other demographic and occupational variables not to incur in omitted variable estimates and to increase the precision of our estimates. We also show that our results are robust when using different model selection techniques.

## Experimental design

### Sample characteristics

A total of 272 participants took part in the study. They were invited to participate through online links posted by the authors, mailing lists, and online announcements distributed on Skype and social networks. The number of participants is within the range of - or larger than - the samples reported in previous adult studies adopting similar experimental protocols (e.g., Iuculano & Butterworth, 2011; Schley & Peters, 2014; Rips, 2012; Sella et al., 2016). No a priori power analysis was conducted for this study.

**Table 1:** Descriptive statistics of the sample

	Variable	Sex (1 = Female)	Age	Years of formal education	Income	Math test score	Shopping scale	Total observations
Condition								
ALL	Mean	0.46	38.2	17.2	5.9	0.76	0	271
	St. Dev.	0.50	9.5	2.8	2.6	0.24	0.79	
	Min	0	23	5	1	0	-0.97	
	Max	1	74	21	11	1	4.95	
	Number of answers	259	260	256	246	271	265	
Money Fuzzy	Mean	0.50	38.9	17.1	5.5	0.75	0	57
	St. Dev.	0.50	11.6	3.0	2.5	0.24	0.68	
	Min	0	23	5	1	0	-0.87	
	Max	1	74	21	11	1	2.21	
	Number of answers	54	53	52	53	57	54	
Money Fixed	Mean	0.56	38.5	17.5	6.0	0.75	-0.05	65
	St. Dev.	0.50	8.9	2.5	2.7	0.23	0.72	
	Min	0	24	13	1	0.11	-0.83	
	Max	1	68	21	11	1	2.76	
	Number of answers	63	63	63	57	65	64	
Number Fuzzy	Mean	0.38	37.7	17.0	6.0	0.76	0	85
	St. Dev.	0.49	9.0	3.1	2.5	0.22	0.75	
	Min	0	25	8	1	0.11	-0.97	
	Max	1	66	21	11	1	3.48	
	Number of answers	82	83	80	77	85	85	
Number Fixed	Mean	0.42	38.1	17.1	6.1	0.76	0.05	64
	St. Dev.	0.50	8.9	2.7	2.8	0.26	0.99	
	Min	0	25	13	1	0	-0.83	
	Max	1	65	21	11	1	4.95	
	Number of answers	60	61	61	59	64	62	
K-W test	z-statistics	5.30	0.70	0.77	1.72	0.44	0.87	
	p-value	0.15	0.87	0.86	0.63	0.9313	0.83	

Note The K-W (Kruskall-Wallis) test was run on the null hypothesis that the observations in the four different conditions were generated by the same distribution. The hypothesis concerning any of the considered demographic and behavioural variables is never rejected. *“Math_Test_Score”* is the overall accuracy derived from questions 8–16 in Supplementary Information: Section B (for each question, a score = 1 was assigned to a correct answer, and 0 to an error). *“Shopping_Scale”* is a summative scale derived from four standardized items in our questionnaire – namely, questions 18, 19, 21 and 22 in Supplementary Information: Section C

**Fig. 2:**
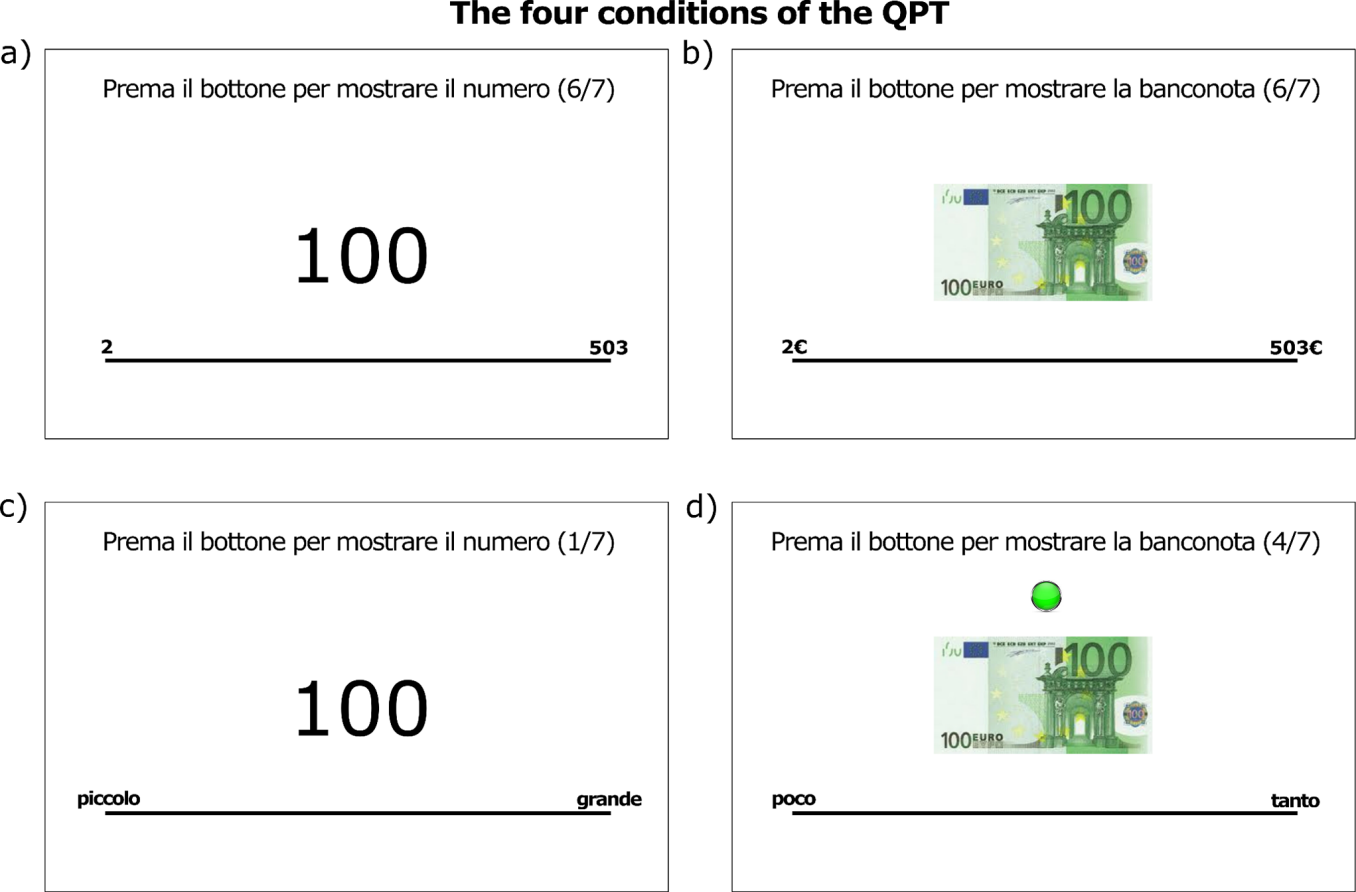
Examples of stimuli presented to participants in the QPT. **a**) *Number fixed* condition. **b**) *Money Fixed* condition. **c**) *Number Fuzzy* condition. **d**) *Money Fuzzy* condition. In each condition, the stimulus presentation was self-paced. Clicking on the green button at the top of the screen resulted in skipping the following trial. “*Piccolo*”, “*grande*”, “*poco*”, and “*tanto*” are the Italian words for “*small*”, ”*large*”, ”*little*”, and ”*much*”, respectively

We excluded one participant over 75, as we thought that the cognitive tasks may have yielded unreliable information in this case. The descriptive statistics of the sample can be found in Table 1. The resulting sample was evenly balanced between males and females (49% females), with a mean age of 38.2 years and a mean formal education of 17.2 years. This means that we oversampled participants with a high level of education in comparison with the general population. Household income was measured through an ordinal scale characterised by 11 categories. Mean and median incomes were located at level 6, corresponding to a gross yearly income between 27,000€ and 32,000€. Participants were randomly assigned to one of the four conditions (*Money Fuzzy*: *N* = 57; *Money Fixed*: *N* = 65; *Number Fuzzy*: *N* = 85; *Number Fixed*: *N* = 64; see Fig. 2 and Sect. Experimental procedure and tasks description for details on the QPT). The random assignment to these four conditions was verified for demographic variables (gender, age, years of formal education, and household income), performance in the math quiz, and a shopping habit scale later used in econometric analysis (see Table 1 and Sect. Influence of socio-economic variables on the performance at the QPT for details and statistical comparisons).

### Experimental procedure and tasks description

Participants performed the study’s tasks in the context of an online survey. The tasks included QPT, a numerical abilities assessment (Supplementary Information: Section B), and a series of questions meant to measure the socio-economic status of the participants and to quantify their spending habits (Supplementary Information: Section C). Participants were randomly assigned to one of the four conditions, so the treatment assignment is between-participant.

In the QPT, participants were presented with a line (length = 646 pixels) in the middle of the screen. Above the line, a quantity appeared in the upper central part of the screen. In the *Number* conditions, this was an Arabic number from the following set: *S*_*N*_ = {5, 10, 20, 50, 100, 200, or 500}. In the *Money* conditions, the same quantities used in *S*_*N*_ were presented to participants as a banknote in Euro currency (stimulus size: 280 × 150 pixels), resulting in the following set: *S*_*M*_ = {5€, 10€, 20€, 50€, 100€, 200€, or 500€}. For each trial, participants were asked to move a cursor with the mouse and then click on one point upon a line, which should correspond to the proposed quantity in the subject’s own assessment. The cursor was always presented under the line, centrally, at the beginning of each trial. Each line had the smallest quantity reported on the left and the largest on the right. Written instructions were given before the task. The extremes of the line differed across four conditions. In the *Fixed range* conditions, the numbers “2” and “503” were placed close to the left and right extremes, respectively. In the *Fuzzy range* conditions, the Italian for the words “*small*” and “*large*” (i.e., “*piccolo*” and “*grande*”, respectively) in the *Number* conditions, and “*little*” and “*much*” (i.e., “*poco*” and “*tanto*”, respectively) in the *Money* condition, were placed on the left and the right of the line, respectively. There were four conditions: *Number Fixed*,* Money Fixed*,* Number Fuzzy*,* and Money Fuzzy* (see Fig. 2).

Each quantity was presented in a random order once, for a total of seven stimuli. All stimuli were then administered a second time, again in a randomized order, to test for the possibility of “learning” effects, i.e., to check whether individuals rely on different representations after having acquired experience with the task. We call the first set of trials *Test* and the second set *Retest*. Participants were then asked to respond to nine questions requiring them to compare the size of pairs of fractions, convert quantities across different formats, and compute the expected outcome values of a variable in different contexts. This test was adapted from Peters et al. (2006). The overall accuracy percentage was computed across the nine questions, reported in Table 1. This series of questions is presented in the Supplementary Information: Section B.

Finally, participants were asked to provide information regarding their economic status (e.g., annual income) as well as their spending habits (e.g., estimates of the amounts spent for buying durable and non-durable goods). This series of questions is presented in the Supplementary Information: Sections A and C (see also Table 1).

### Instrument

The survey was hosted on a server belonging to an academic institution where one of the authors was working during data collection, but with which the author has no contact anymore. The survey was a self-developed tool developed using PHP (https://www.php.net) and jQuery (https://jquery.com) software. In particular, jQuery tracked mouse position over the images and recorded participants’ responses, including Likert scale inputs.

## Results

Our analysis is organized as follows. Firstly, we analyzed the QPT results by contrasting the presence of logarithmic or linear representations in the different conditions at both the group and individual levels (Sect. QPT performance at the group level and individual level). Following the results of these analyses, we then focused on the difference between money and number representations (Sect. Comparison of the numerical and money conditions for individual stimuli in the QPT). Finally, we analyzed the impact of socioeconomic factors and mathematical abilities on the degree of linearity in the QPT (Sect. Influence of socio-economic variables on the performance at the QPT). The open-source R software (R Core Team, 2024; Version 4.4.0) and the packages *dplyr* (Wickham, François, Henry, & Müller, 2023), *tidyr* (Wickham et al., 2024), and *ggplot2* (Wickham, 2016) were used for data preprocessing and visualisation. The software R and JASP (JASP Team, 2024; Version 0.18.3) were used for statistical analyses reported in Sect. QPT performance at the group level and individual level and Comparison of the numerical and money conditions for individual stimuli in the QPT; the software STATA was used for the econometric analysis reported in Sect. Influence of socio-economic variables on the performance at the QPT. The dataset and the reproduction codes are available at the repository: https://osf.io/8h7jr/.

### QPT performance at the group level and individual level

Our observed variable was the position of the response on the line, measured as the percentage of absolute error (PAE) from its left extreme, which corresponded to the smallest quantities in all conditions. This would be obtained if a participant located all stimuli in the QPT at intervals whose length equals the difference between the stimulus and the extreme of the line in proportion to the total length of the line. For instance, a perfectly linear response would imply that stimulus 5 should be placed in the QPT at 0.60% of the total length of the line starting from the left extreme (because 0.0060 = (5 − 2)/(503-2). The stimulus 10 should be placed at 1.99% of the total length of the line starting from the left extreme (because 0.0199 = (10 − 2)/(503-2), etc. Following the statistical approach of previous studies (Opfer, 2003), to determine the presence of logarithmic or linear performance at the group level, we fitted both a linear and a logarithmic interpolation of the median PAE of the group (dependent variable) according to stimulus quantity (either *S*_*N*_ or *S*_*M*_: predictor) for each of the four conditions. We then compared residuals’ absolute values between the logarithmic and the linear interpolation to identify which model best fitted the results.

**Fig. 3:**
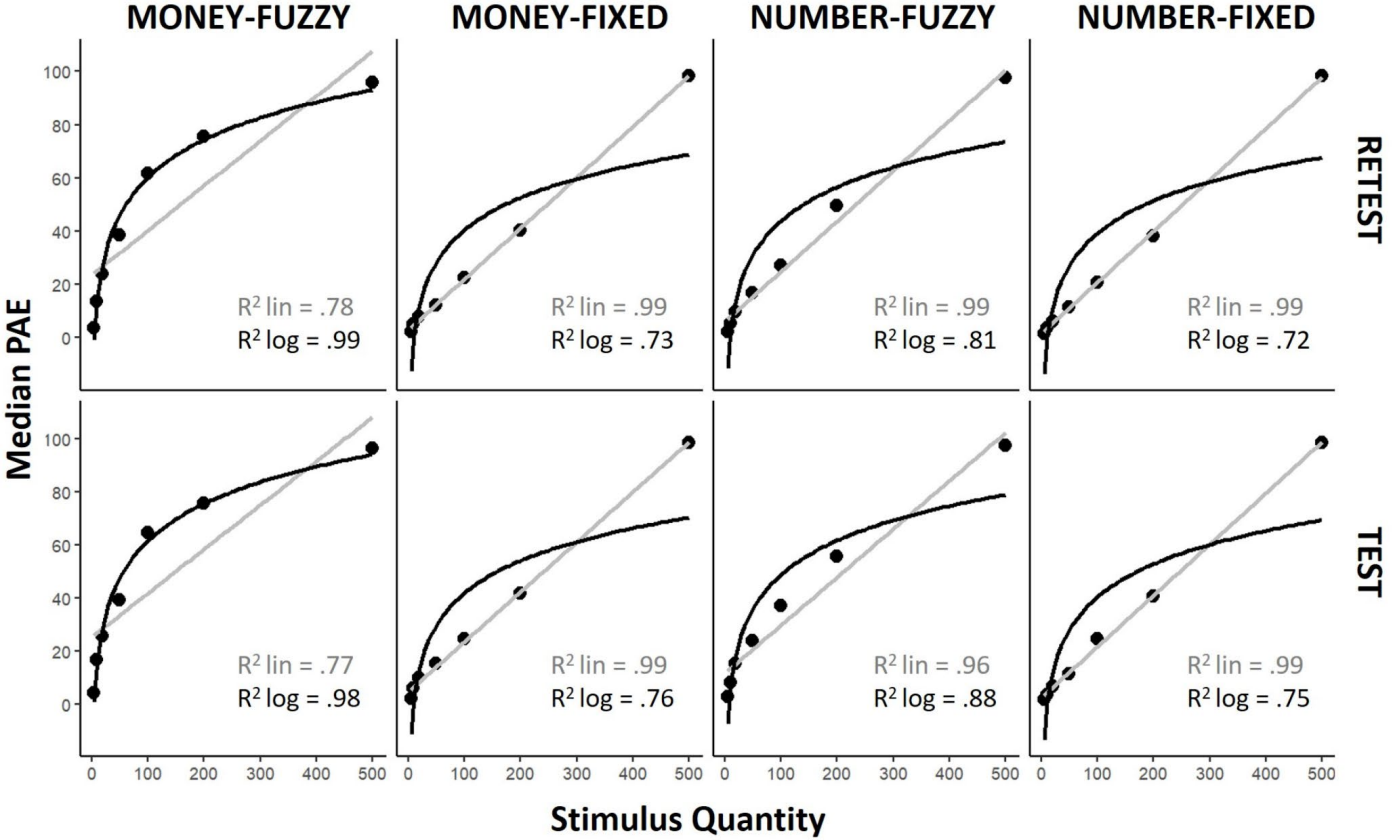
Median response in the QPT at the group level for each quantity and condition is plotted (black dots). The fits of linear (grey line) or logarithmic (black line) interpolation are also plotted, and the corresponding R^2^ is reported

*Test* and *Retest* were analyzed separately. We acknowledge that the results were compatible with the results obtained by putting together the *Test* and *Retest* (See Supplementary Information); nonetheless, we report the results from the *Test* and *Retest* separately to highlight potential differences induced by the repetition of the task. Mean responses for the different conditions are illustrated in Fig. 3. In the *Test*, data in the *Fixed* range conditions were best fitted by linear interpolation, both in the *Number* (R^2^ lin-fit = 0.998; R^2^ log-fit = 0.75; comparison of residuals: t(6)=-4.1; *p* < 0.01) and in the *Money* conditions (R^2^ lin-fit = 0.997; R^2^ log-fit = 0.76; comparison of residuals: t(6)=-3.8; *p* < 0.01). On the contrary, the logarithmic function best fitted the data in the *Money Fuzzy* condition (R^2^ lin-fit = 0.77; R^2^ log-fit = 0.98; comparison of residuals: t(6) = 2.9; *p* < 0.05), while no difference emerged between linear and logarithmic fit in the *Number Fuzzy* condition (R^2^ lin-fit = 0.96; R^2^ log-fit = 0.88; comparison of residuals: *p* > 0.1). Results were similar in the *Retest*, except for the *Number Fuzzy* condition, where a linear trend emerged: the linear function best fitted the data in the *Fixed* range conditions, both for *Number* (R^2^ lin-fit = 0.999; R2 log-fit = 0.73; comparison of residuals: t(6)=-4.0; *p* < 0.01) and for *Money* conditions (R^2^ lin-fit = 0.999; R2 log-fit = 0.73; comparison of residuals: t(6)=-4.0; *p* < 0.01); the logarithmic equation best fitted the data in the *Money Fuzzy* condition (R^2^ lin-fit = 0.78; R^2^ log-fit = 0.99; comparison of residuals: t(6) = 3.3; *p* < 0.05); while there was no difference between linear and logarithmic fit in the *Number Fuzzy* condition (R^2^ lin-fit = 0.999; R^2^ log-fit = 0.73; comparison of residuals: t(6)=-4.04; *p* < 0.01). These results support our hypotheses *H*_*1*_ and *H*_*2*_ (see Sect. Introduction).

Individuals’ responses in the task were then fitted using logarithmic or linear equations, using the quantities of the stimulus, i.e. the quantities belonging to either *S*_*N*_ or *S*_*M*_ as predictors. This fit was not provided for participants with four or more missing values in either the *Test* or the *Retest* (*N* = 4). Additionally, five participants with partial data were also excluded following a quality check of the data or problems in the fitting. Both in *Test* and *Retest*, the fit of the linear model appears to be higher in the *Fixed* conditions in comparison to the *Fuzzy* conditions. Fixed conditions *Test*: *Number*: mean R^2^ lin-fit = 0.96; mean R^2^ log-fit = 0.76; Money: mean R^2^ lin-fit = 0.93; mean R^2^ log-fit = 0.77; Fixed conditions *Retest*: *Number*: mean R^2^ lin-fit = 0.98; mean R^2^ log-fit = 0.74; Money: mean R^2^ lin-fit = 0.97; mean R^2^ log-fit = 0.74). *Fuzzy* conditions *Test*: *Number*: mean R^2^ lin-fit = 0.83; mean R^2^ log-fit = 0.80; Money: mean R^2^ lin-fit = 0.73; mean R^2^ log-fit = 0.89; Fuzzy conditions *Retest*: *Number*: mean R^2^ lin-fit = 0.86; mean R^2^ log-fit = 0.79; Money: mean R^2^ lin-fit = 0.75; mean R^2^ log-fit = 0.90; see Sect. Influence of socio-economic variables on the performance at the QPT for statistics). The results for the logarithmic model are symmetric.

When considering the highest R^2^ as indicating the best fitting model for each participant, we observed that participants were highly consistent, as indicated by the high proportion of individuals who did not change representation (*Money Fuzzy*: 87%; *Money Fixed*: 86%; *Number Fuzzy*: 75%; *Number Fixed*: 92%).

### Comparison of the numerical and money conditions for individual stimuli in the QPT

**Fig. 4:**
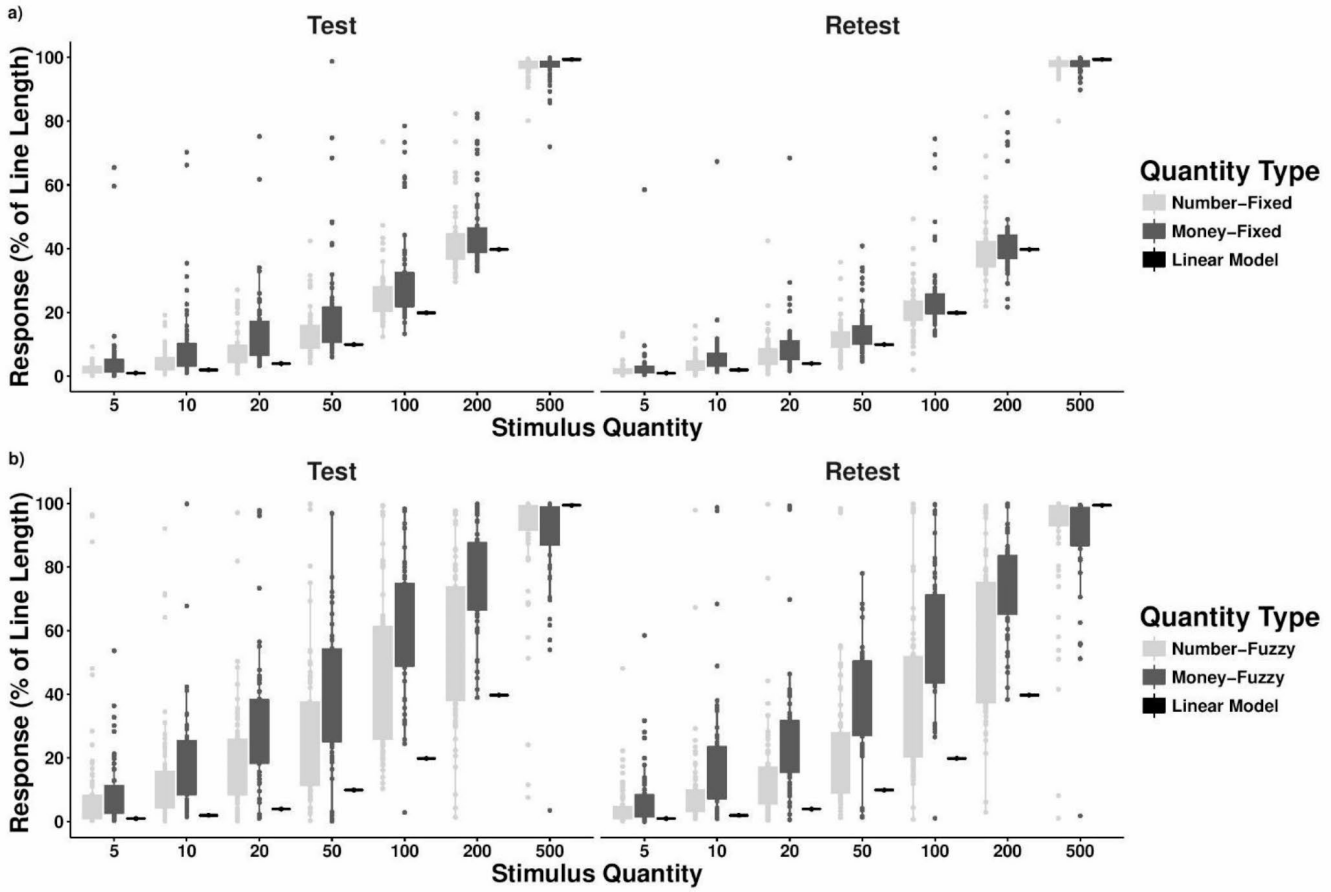
Panels a (*fixed* range) and b (*fuzzy* range) report box plots for the distribution of the responses in the QPT for the different quantities (5, 10, 20, 50, 100, 200, 500), conditions (*Number* in light grey, *Money* in dark grey), and phase (*Test* on the left, *Retest* on the right). The hypothetical responses, if an individual follows a perfectly linear representation, are also reported (Linear Model, in black). The box upper (lower) hinge identifies the 75th (25th) percentile and the circles identify outside values

After having assessed the overall fitness of the linear vs. logarithmic models, we analysed participants’ PAE. We divided the analysis between *Fixed* and *Fuzzy* conditions. In both cases, we found that participants deviated significantly more from the perfectly linear response for *Money* than for *Number*. In particular, they systematically overestimated the stimuli in *Money* compared to *Number* conditions, and this is in line with the observation that representations have a more pronounced concavity in the former than in the latter case. Figure 4 reports the distribution of responses in relation to the linear representation for each stimulus and condition.

As shown by Fig. 4, in the *Fixed* conditions, larger overestimation in the *Money* than *Number* condition occurred for all stimuli in both the *Test* (Two-sided Mann-Whitney tests: Q5: z = -2.740, *p* < 0.01; Q10: z = -3.526, *p* < 0.001; Q20: z = -3.513, *p* < 0.001; Q50: z = -2.930, *p* < 0.005; Q100: z = -1.915, *p* = 0.06; Q200: z = -1.780, *p* = 0.08; Q500: z = -0.334, *p* = 0.73) and the *Retest* (Q5: z = -2.513, *p* = 0.012; Q10: z = -3.622, *p* < 0.001; Q20: z = -2.319, *p* = 0.02; Q50: z = -1.886, *p* = 0.06; Q100: z = -2.021, *p* = 0.04; Q200: z = -1.742, *p* = 0.08; Q500: z = − 0.22, z = 0.82). A similar pattern occurred in the *Fuzzy* conditions. Larger overestimation occurred in the *Money* than the *Number* condition for all stimuli except 500 in both the *Test* (Two-sided Mann-Whitney tests: Q5: z = -1.674, *p* = 0.09; Q10: z = -3.330, *p* < 0.001; Q20: z = -3.933, *p* < 0.001; Q50: z = -3.305, *p* < 0.01; Q100: z = --3.934, *p* < 0.001; Q200: z = -5.051, *p* < 0.001; Q500: z = 0.83.2, *p* = 0.4) and the *Retest* (Q5: z = -2.42, *p* = 0.03; Q10: z = z = -4.602, *p* < 0.001; Q20: z = -5.383, *p* < 0.001; Q50: z = -5.43, *p* < 0.001; Q100: z = -5.512, *p* < 0.001; Q200: z = -4.344, *p* < 0.001; Q500: z = 1.866, z = 0.06).

These results indicate that monetary quantities were overall overestimated compared to numbers, supporting *H*_*3*_.

### Influence of socio-economic variables on the performance at the QPT

The hypothesis we then tested concerns the impact of spending habits on the cognitive representation of quantities, presented either in numerical format or as monetary values. This builds on the idea, put forward by e.g. Ungemach et al. (2011) and Vlaev et al. (2011), that spending habits influence individuals’ numeric and money representation. Our dependent variable to test for this hypothesis was the result of a test of linearity over the participants’ placement of the experimental stimuli described above. More specifically, we used the R^2^ resulting from fitting a linear model to the placements of single subjects. This method has been used by previous studies (e.g., Sella et al., 2016). The higher the R^2^ values, the closer the participant’s representation of quantities to the linear model. We called this variable “*Linearity*”. *Linearity_Test* and *Linearity_Retest* refer, respectively, to the results of the *Test* or the *Retest*. Our primary observed variable measured individual spending habits, called *“Shopping_Scale”*. We derived such a scale from subjects’ answers to four items in our questionnaire – namely, questions 18, 19, 21 and 22 in the Questionnaire (see Supplementary Information: Section C). Such items asked participants to state how much they would typically spend on goods purchased and how much they spent the last time they went shopping. These two questions were replicated for both everyday use items (such as bread, drinks, newspaper) and durable goods (e.g., clothing). *Shopping_Scale* is a simple summative scale of the four items after they have been standardised. The Cronbach’s alpha of the scale is 0.69, confirming the relatively good reliability of the scale. Descriptive statistics are reported in Table 1. The higher the scale, the larger the sums of money a subject spends. Even in this case, a Kruskall-Wallis test confirms the exogeneity of the assignment to the four different conditions of this variable (see Table 1 for details).

Tables 2 and 3 report the results of Ordinary Least Square (OLS) regressions fitted to the *Linearity_Test* R-Squared value of the linear interpolation of a subject’s placement in the *Test* (Table 2) and *Linearity_Retest* in the *Retest* phase (Table 3). The first six regressions were used as independent variable dummies to identify the experiment conditions, the omitted category being the *Number_Fix* condition. The first model looked at demographic factors, including a large array of variables measuring a subject’s socio-economic status (SES). SES characteristics are very likely to influence shopping spending habits, especially with respect to the amount of money being spent, and thus, they might confound the relationship we wanted to investigate. We used dummies to identify participants’ self-reported income categories. The original question allowed for 11 possible categories^2^. In the regression, we combined some of these categories to form a four-level income scale, including *“Low”*, *“Medium-Low”*, *“Medium Hig”*, and *“High”* income levels. *Low_income* is the residual category.

We also included a measure of the social status of a subject’s occupation, based on Ganzeboom and Treiman (1996) classification of the social status associated with a certain occupation derived from international surveys. This scale has been validated internationally and is often used to classify occupations in social research. Occupations were divided into *“High social status”* and *“Medium-Low social status”*. Participants who were outside of the labour force - either because they were unemployed or because they were students or housewives/husbands – formed the residual category. We also included dummy variables to identify those working in education or the financial sector. We also controlled for a subject’s education through a dummy variable identifying participants having attained a Master or a Ph.D. Introducing a finer identification of educational attainments does not lead to appreciable changes in our results. Such a vector of variables affecting participants SES was probably more comprehensive than what is usually done in this type of analyses. Still, we wanted to be cautious in controlling for as many possible confounding factors as possible. Finally, we controlled for a subject’s age (linearly and with a squared term), gender, civil status (married or cohabiting with a partner, as opposed to separated, divorced, widowed, or not in a relationship), and geographical origin within the Italian territory (see notes to Supplementary Information: Table SI1 for a detailed description of SES variables).

**Table 2:** OLS regression analysis of test task

	(1)	(2)		(3)		(4)		(5)		(6)		(7)	(8)	
Dependent variable	*R*-squared linear fit: test													
Cond_Number_fuzzy	-0.119***	-0.118***		-0.148***		-0.116***		-0.117***				-0.105		
	(0.0246)	(0.0245)		(0.0543)		(0.0239)		(0.0244)				(0.0985)		
Cond _Money_fix	-0.0260	-0.0249		-0.0434		-0.0179		-0.0185				0.118*		
	(0.0251)	(0.0250)		(0.0485)		(0.0252)		(0.0256)				(0.0613)		
Cond _Money_fuzzy	-0.215***	-0.216***		-0.211***		-0.216***		-0.216***				-0.194		
	(0.0242)	(0.0236)		(0.0402)		(0.0227)		(0.0230)				(0.119)		
Income_med_low	0.00118					-0.00527		-0.00428		-0.0116		-0.00735	-0.0130	
	(0.0384)					(0.0367)		(0.0371)		(0.0424)		(0.0352)	(0.0417)	
Income_med_high	0.0655**					0.0469*		0.0469*		0.0551*		0.0491*	0.0564*	
	(0.0299)					(0.0274)		(0.0276)		(0.0305)		(0.0276)	(0.0301)	
Income_high	0.0191					-0.00234		-0.00256		0.00246		-0.00132	0.00300	
	(0.0326)					(0.0318)		(0.0321)		(0.0355)		(0.0312)	(0.0349)	
Income_medium_&_high		0.0431*		0.0271										
		(0.0239)		(0.0286)										
Income_X_Number_fzy				0.0453										
				(0.0617)										
Income_X_Money_fix				0.0273										
				(0.0532)										
Income_X_Money_fzy				-0.0125										
				(0.0499)										
Shopping_scale						0.0249***		0.0217***		0.0218**		0.0257***	0.0197*	
						(0.00896)		(0.00832)		(0.0103)		(0.00895)	(0.0102)	
Math_test_score_perc							0.0863**		0.0857**		0.0726	0.155***		0.147**
							(0.0414)		(0.0421)		(0.0501)	(0.0561)		(0.0673)
Shopping_Scale_X_Number_fzy									0.00786					
									(0.0168)					
Shopping_Scale _X_Money_fix									0.00984					
									(0.0215)					
Shopping_Scale _X_Money_fzy									-0.00685					
									(0.0279)					
Cond_Money											-0.0488**			0.0575
											(0.0227)			(0.0845)
Shopping_Scale _X_Money											-0.00743			
											(0.0205)			
Maths _X_Number_fzy												-0.0130		
												(0.111)		
Maths _X_Money_fix												-0.159**		
												(0.0691)		
Maths _X_Money_fzy												-0.0238		
												(0.131)		
Math_ab_X_Money														-0.123
														(0.0935)
Constant	1.041***		1.068***		1.102***		1.003***		1.006***		0.757***	0.949***		0.708***
	(0.141)		(0.137)		(0.142)		(0.148)		(0.150)		(0.178)	(0.144)		(0.170)
Demographic controls	Yes		Yes		Yes		Yes		Yes		Yes	Yes		Yes
Money conditions (aggregate)	-0.12***		-0.12***		-0.11		-0.12***		-0.11***			0.029		
	(0.040)		(0.040)		(0.072)		(0.039)		(0.039)			(0.152)		
Observations	206		206		206		206		206		206	206		206
R-squared adj.	0.258		0.252		0.245		0.286		0.276		0.0267	0.292		0.0379

**Notes**: The dependent variable is the R^2^ of the linear interpolation of individual observations in the *Test* phase of the QPT. *Cond _Number_fuzzy*,* Cond _Money_fix*, and *Cond _Money_fuzzy* are dummy variables identifying three of the four conditions (*Treat_Number_fix* being the omitted category). *Income_med_low*,* Income_med_high*, and *Income_high* identify different income categories, as per answers to Question 6 in the questionnaire (see Supplementary Information, Section A). In particular, *Income_med_low* identify categories 4–6, *Income_med_high* identify categories 7–9, and *Income_high* identify categories 10 and 11. *Income_medium_&_high* identify income categories 7–11. *Income_X_Tr.* are interaction effects between the Income variable and the three conditions used in the analysis. *Shopping_scale* is the summative scale of answers to Questions 18, 19, 21, and 22 of the Questionnaire (Section C). *Math_test_score_perc* is the score in the 9-item test of computational abilities (see Questions 8–16 in Supplementary Information, Section B). *Shopping_Scale_X_Cond* is the interaction between *Shopping_scale* and three Condition dummies. *Maths_X_Cond.* is the interaction between *Math_test_score_perc* and three Condition dummies. *Cond_Money* is a dummy variable identifying the two Money Conditions. *Shopping_Scale__X_Money* is an interaction term between *Shopping_scale* and *Cond_Money*. Likewise, *Maths__X_Money* is an interaction term between *Math_test_score_perc* and *Cond_Money*. Other demographic controls are included and reported in Supplementary Information: Table SI1. The line denoted *Money conditions (aggregate)* reports results on Wald tests on coefficients of condition to test whether the dependent variable was significantly different in the two Money conditions than in the two Number conditions. More precisely, the test is on the null H_0_ = β_Cond _Money_fix_ + β_Cond _Money_fuzzy_ - β_Cond_Number_fuzzy_ = 0. An OLS model has been fitted. Reported in parenthesis are Huber-White standard errors robust to heteroschedasticity

**Table 3:** OLS regression analysis of the retest task

Dependent variable	(1)	(2)	(3)	(4)	(5)	(6)	(7)	(8)
	*R*-squared linear fit: retest							
Cond_Number_fuzzy	-0.112***	-0.111***	-0.126***	-0.111***	-0.112***		0.0565	
	(0.0242)	(0.0247)	(0.0426)	(0.0243)	(0.0249)		(0.0449)	
Cond _Money_fix	0.00109	0.00141	-0.0293	0.00206	0.00161		-0.0216	
	(0.0147)	(0.0140)	(0.0391)	(0.0143)	(0.0142)		(0.0843)	
Cond _Money_fuzzy	-0.206***	-0.204***	-0.155***	-0.207***	-0.207***		-0.0733	
	(0.0266)	(0.0266)	(0.0252)	(0.0268)	(0.0270)		(0.0525)	
Income_med_low	0.0241			0.0233	0.0243	0.0164	0.0304	0.0170
	(0.0311)			(0.0304)	(0.0308)	(0.0330)	(0.0295)	(0.0329)
Income_med_high	0.00114			-0.00207	-0.00208	0.00879	0.00148	0.00836
	(0.0274)			(0.0247)	(0.0252)	(0.0288)	(0.0241)	(0.0288)
Income_high	0.0269			0.0240	0.0236	0.0315	0.0271	0.0309
	(0.0253)			(0.0241)	(0.0242)	(0.0276)	(0.0238)	(0.0278)
Income_medium_&_high		0.00112	0.00530					
		(0.0205)	(0.0151)					
Income_X_Number_fzy			0.0205					
			(0.0515)					
Income_X_Money_fix			0.0449					
			(0.0424)					
Income_X_Money_fzy			-0.0950*					
			(0.0504)					
Shopping_scale				0.00233	-0.00108	-0.00203	0.00130	-0.00349
				(0.0115)	(0.00828)	(0.0153)	(0.0110)	(0.0135)
Math_test_score_perc				0.0151	0.0156	-0.0100	0.0737*	-0.0437
				(0.0408)	(0.0412)	(0.0410)	(0.0395)	(0.0412)
Shopping_Scale_X_Number_fzy					0.00684			
					(0.0275)			
Shopping_Scale _X_Money_fix					0.0103			
					(0.0121)			
Shopping_Scale _X_Money_fzy					-0.00348			
					(0.0394)			
Cond_Money						-0.0330		-0.0866
						(0.0246)		(0.0658)
Shopping_Scale _X_Money						-0.00383		
						(0.0243)		
Maths _X_Number_fzy							-0.195***	
							(0.0643)	
Maths _X_Money_fix							0.0291	
							(0.0876)	
Maths _X_Money_fzy							-0.153**	
							(0.0655)	
Math_ab_X_Money								0.0624
								(0.0742)
Demographic controls	Yes	Yes	Yes	Yes	Yes	Yes	Yes	Yes
Constant	1.019***	1.005***	1.078***	1.012***	1.015***	0.765***	0.964***	0.790***
	(0.168)	(0.164)	(0.171)	(0.172)	(0.171)	(0.185)	(0.172)	(0.188)
Money (Aggregate)	-0.093**	-0.091**	-0.059	-0.093**	-0.094**		-0.151	
	(0.040)	(0.040)	(0.059)	(0.041)	(0.041)		(0.099)	
Observations	209	209	209	209	209	209	209	209
R-squared adj.	0.236	0.238	0.250	0.229	0.217	0.261	0.247	0.231

**Notes**: The dependent variable is the R^2^ of the linear interpolation of individual observations in the *Retest* phase of the QPT. See notes in Table 2

Regressions 1 and 2 confirmed the significant effects of the experimental manipulation detailed in Sect. QPT performance at the group level and individual level and Comparison of the numerical and money conditions for individual stimuli in the QPT. Participants were significantly closer to a linear representation of quantities, and thus, their R-squared in *Linearity_Test* is significantly higher in *Fixed* than *Fuzzy* conditions. Moreover, within *Fuzzy* conditions, individuals’ representation was significantly closer to the linear model in the *Number Fuzzy* than in the *Money Fuzzy* condition (Two-tailed Wald test: β=-0.091; t= -2.98; *p* = 0.003). A similar pattern was observed in the comparison between *Money Fixed* and *Number Fixed*, but the two coefficients were not significantly different from each other (Wald test: β=-0.026; t= -1.05; *p* = 0.294). In the aggregate, Money conditions were significantly less likely to be represented by a linear representation, as emerges from two-tailed Wald tests on differences across conditions involving all the four coefficients (β=-0.12; t= -3.09; *p* = 0.002; see footnote to Table 2 for details on the test).

Additionally, among the SES variables, *Medium_High_Income* significantly affected the *R-squared_linear_fit Test*. This effect was confirmed, albeit weakly, by combining the two higher-income categories and two lower-income categories (See Table 2, column 2). This means that, controlling for other variables, the higher the participant’s income, the closer the numerical representation is to linear. No other demographic variable was significant (see Supplementary Information: Table SI1). Regression 3 in Table 2 examined whether income exerts differential effects in the *Number* or *Money* conditions, introducing four interaction terms between income (measured with a dummy variable identifying the above-the-median income categories) and the four conditions. However, no significant effect was detected. This may be due to the limited number of observations per condition. However, interaction terms were not significant even combining *Fixed* and *Fuzzy* conditions together (not reported). We concluded that participants’ income does not show a differential effect in the *Number* vs. *Money* conditions.

The following regressions included *Shopping_Scale* and “*Math_Test_Score*”, the latter measuring participants’ ability in mathematical and statistical computations (see Table 1) into the model. Both variables exert significantly positive effects on the *R-squared_linear_fit_Test*. In particular, the effect of *Shopping_Scale* was significant (β = 0.024; t = 2.64; *p* = 0.009). This unambiguously supports *H*_*4*_, i.e. the hypothesis that individuals’ spending habits, and specifically their familiarity with handling large quantities of money, sharpens the precision of their representation of numerical and monetary quantities. The effect of *Math_Test Score* was also significant (β = 0.085; t = 2.07; *p* = 0.040), supporting *H*_*5*_. We also analyzed whether *Shopping_Scale* exerted differential effects in the *Money* treatments vs. the *Number* treatments. Regression 5 and 6 in Table 2 tested the interaction of *Shopping_Scale* with the conditions dummy or a dummy combining *Fixed* and *Fuzzy* conditions for *Money* and *Number* conditions. No interaction effect emerged, allowing us to conclude that *Shopping_Scale* exerts similar effects in *Money* and *Number* conditions. Finally, regressions 7 and 8 checked for differential effects of *Math_Test_Score* across conditions. A significantly lower effect of ability in math tests in the *Money_fix* condition compared to the *Number_fix* condition emerged. All other comparisons were not significant. Combining *Fixed* and *Fuzzy* conditions together results in *Math_Test_Score* being significant only in the *Number* conditions but not in the *Money* conditions (Table 2, column 8). This suggests that mathematical ability exerted overall a larger effect when participants dealt with number representations rather than money representations.

We replicated this analysis for the *Retest* task. While condition effects were virtually unaltered in *Retest* compared to *Test* (Table 3, columns 1–2), this was not the case for income, *Shopping_Scale* and *Math_Test_Score*, which were no longer predictive of *Linearity Retest*. A plausible interpretation of these results is that the effect of both *Shopping_Scale* and *Math_Test_Score* is limited to the tasks where participants do not have prior experience with the task and where their cognitive functioning relies on previously acquired heuristics re-adapted to the laboratory task; in *Retest* task, on the contrary, participants rely on their prior experience of the task and this changes their heuristics as they somehow ‘learn’ from their previous task and become more precise. Once again, no SES variables are significant, except for a weakly significant effect of having high or intermediate employment status compared to having low status.

In order to better appreciate the differences between the Test and Retest tasks, we performed OLS regression analysis on panel data in which data from the Test and Retest tasks were pooled. We refer the reader to Table SI1 and the note for more details on the econometric model. We report the values separately for test-retest in the Supplementary Information. The interaction term between the Retest and each condition coefficient is not significantly different from zero, thus suggesting that condition effects were not dissimilar in the *Test* and *Retest*. In fact, coefficients for the three conditions have approximately the same size and levels of significance in the *Test* and *Retes*t, confirming the robustness of our main results. The coefficient for Retest_Response, identifying decisions from the Retest, is positive and significantly different from zero in the basic model (*p* = 0.014, Supplementary Information: Table SI2, column 1) as well as all other models (see Supplementary Information: Table). This suggests that, while responses to the task tended to follow a linear representation more often in *Test* than *Retest*, differences between conditions remained similar. Even if the null hypothesis that the effect of *Shopping_Scale* in *Test* and *Retest* is not rejected (*p* = 0.22), *Shopping_Scale* is found to have a significant effect on responses only in the *Test* response (*p* = 0.031) but not in the *Retest* response (*p* = 0.45). The coefficient for *Shopping_Scale* is now at the margins of statistical significance (*p* = 0.061; *p* = 0.108, respectively; Table SI1, column 4).

We included every demographic or occupational factor in the main regression model to avoid biased estimates with omitted variables. Since most of these covariates are not significant, one may wonder whether the results over *Shopping_Scale* remain stable in Test when some or all of these control variables are omitted. For this reason, we applied the LASSO model selection technique proposed by Jolliffe et al. (2003). This technique tests various specifications of the model, alternating, in turn, some of the covariates, yielding a model “optimal” for predictive purposes. Typically, this model selection technique prescribes dropping a large number of covariates. We applied two selection techniques: cross-validation (the default in Stata) and adaptive. Results of the model concerning the selected variables are available in Table SI4 of the Supplementary Information. The Cross-Validation and Adaptive techniques yield models with eight and ten covariates, respectively, out of the initial sixteen. The fact that the *Shopping Scale* and *Math_test_score* were selected under both techniques, even if no restriction on the variables to include was given, confirms the high predictive capacity of these variables. The related coefficients are positive and strongly significantly different from 0 (*p* = 0.014 and *p* = 0.002 for the *Shopping Scale* in the two models, respectively; *p* = 0.003 and *p* = 0.007 for *Math_test_score*, respectively).

## Discussion

In the present study, we compared potential differences between the way quantities are represented when related to monetary values vs. when they are presented in abstract (with Arabic numerals) format. Moreover, we investigated the mediating role of individual variables, such as socioeconomic status and buying habits. To reach our aims, we used a variant of the number-to-position task, a well-known paradigm frequently used to investigate accuracy in number line estimation as a function of several variables, such as age and mathematical skills (Siegler & Opfer, 2003; Schneider et al., 2018). Participants had to position on a line quantities expressed either in numerical format (*Number* condition, e.g., 50) or as monetary values (*Money* condition, e.g., 50€). The extremes of the line consisted of specific values (*Fixed* condition, i.e. “2€”___”503€”) or of unspecified concepts of magnitude (*Fuzzy* condition, e.g., “little”__” a lot”). In the following paragraphs, we summarise and discuss our main findings.

First, we found that when bounds were fixed, a linear representation best fitted the data for both monetary and numerical formats compared to a logarithmic representation. However, when the bounds were fuzzy, significant deviations from a linear representation (larger PAE) emerged, with most of the participants’ representation being best fitted by a logarithmic pattern in the *Money-Fuzzy* condition. This result suggests that the presence of monetary values in the context of fuzzy references triggered different processes, likely related to the use of money, in turn leading to a more compressed representation. To our knowledge, this is the first study investigating the effects of fuzzy ranges in the estimation of quantities with the number line task. It is interesting to note that the logarithmic mapping observed with money and fuzzy ranges has similarities with the performance of children and adults in processing nonsymbolic quantities (e.g., Dehaene, 2003; Izard & Dehaene, 2008). It has been suggested that humans – as well as different animal species – are equipped with an approximate number system, defining the ability to perceive and discriminate quantities in the environment, similar to a *number sense* (Dehaene, 1997). According to classic models of numerical development, the acquisition of symbolic number knowledge is built upon this approximate representation of quantities, even if there has been no agreement concerning the degree of overlap between symbolic and nonsymbolic processes in adulthood (e.g., Lyons et al., 2011). Beyond this debate, it is clear that the two systems co-exist and are preferentially activated in the function of task requirements. In this sense, one possible explanation for the differences observed among conditions is that when placing money in a fuzzy range, participants tend to rely more on an approximate, basic and elemental sense of magnitude.

Second, even if most participants’ representations can be overall “classified” as linear, except in the *Money-Fuzzy* condition, we find significant differences in the way abstract numeric and money quantities are represented. Specifically, the analysis of responses to individual stimuli reveals a larger PAE in *Money* than in *Number* conditions for both fixed and fuzzy extremes. Such a pattern of performance leads to systematic overestimations of the stimuli. This finding further corroborates the active processing of money information. We further speculate that a less abstract quantity like money might be represented in a way which is not the purely symbolic, abstract representation but resembles the one shown for non-symbolic quantities. Importantly, the representation of money must necessarily include some aspects related to its value. Specifically, quantities of money are processed in everyday life in different concrete experiences of profit and consumption, usually involving price judgments or estimation, decision-making, and emotional experiences (e.g., Hirabayashi et al., 2021; Norton et al., 2012; Oishi et al., 2023). It seems then reasonable to assume that, even when quantities of money do not refer to concrete contexts, they convey information beyond the pure representation of abstract quantities.

An alternative explanation can be found in perceptual effects. In our experiment, numerical stimuli were physically smaller than banknotes and this might have led to a perceptual bias influencing performance. Even if we cannot exclude this possibility, we also underline that our between-participants design should have minimised this potential confound. Moreover, this perceptual difference cannot explain the difference between fuzzy and fixed conditions. Also, it has been shown that presenting monetary stimuli in their actual colour, size, and design helps in the processing of their value (Ojedo & Macizo, 2023). In this study, the colour and design of banknotes were the same as in authentic contexts, while the size was constant and not incongruent with authentic banknotes. In this sense, the impact of perceptual features of monetary stimuli has been minimised.

Crucially, we observed that cognitive and socioeconomic aspects affected such representations. A strong effect of spending habits on the degree of linearity of performance was observed, whereby persons accustomed to spending larger sums of money in their shopping also tended to be more accurate – i.e. adhering more closely to a linear model - in their representations. Interestingly, as opposed to our hypothesis, this pattern was similar in the money and number conditions. In contrast, we were expecting a larger influence in the money condition. Finally, the results of our math test better accounted for performance in the *Number* than in the *Money* condition. The basic assumption of decreasing marginal utility provides a useful framework to interpret the finding that individuals less used to dealing with monetary quantities had a tendency to follow a logarithmic representation. This result further confirms that more extensive practice in dealing with quantities results in effectively implementing strategies to overcome spontaneous representational biases. Importantly, this study has similarities with a previous study by Schley and Peters (2014), who found that the accuracy in representing symbolic number quantities in the number to line task (e.g., 100) predicted performance in a different task (willingness to drive and risky choice paradigms), when the same quantity was presented in monetary format (100 dollars). Interestingly, in their study, it was also observed that numeracy mediates the relation between accuracy in the number line task and money processing, further confirming the partial reliance as well as the independence of the processing of monetary values on numerical knowledge.

Finally, some other methodological considerations should be made. First, in this study, we defined sample size based on consistency with other studies in the same field as ours. When we conducted our study, a-priori power analysis was not yet considered the best practice in determining the sample size of a study. We now recommend this technique for future studies.

Second, we contrasted the different conditions across participants while also avoiding the effects of repeated exposure to the task. Indeed, each stimulus was presented only twice (*Test* and *Retest* phases). Although this allowed us to test only a few quantities, this methodology had the advantage of minimizing the effect of learning and the implementation of copying strategies. It allowed us to measure the different quantity representations more directly than when different conditions, each encompassing several trial repetitions, are adopted.

Third, another methodological consideration concerns the importance of investigating the underlying strategies adopted by the participants to position quantities, which might not necessarily rely on linear or logarithmic mappings. In this study, we compared linear vs. logarithmic mappings because it has been shown that this approach has been shown to capture the performance. Budget and sample limitations prevented us from investigating strategies alternative to the linear or logarithmic model, which participants may use when solving the task under different conditions (fuzzy, fixed) or with different types of quantities (money, numbers). Future studies may focus on these important modelling issues.

## Conclusion

Our study uncovered important differences in the way individuals represent numerical as opposed to monetary values. In line with our hypotheses, the representation of monetary values appears to deviate more from a linear representation, which is consistent with the principle of decreasing marginal utility, a cornerstone of microeconomic theory. We also found that individuals more used to dealing with large sums in real life represent values more closely to a linear model than a logarithmic one. While this study cannot result in any direct recommendation, it goes in the direction of studying the field between numerical cognition and behavioural economics (e.g., Guido et al., 2016; Macizo & Ojedo, 2018; Schley & Peters, 2014). Many human behaviours and choices are based on the manipulation of quantities: any bias in their representation might result in suboptimal strategies being implemented. Consequently, integrating behavioural economics with experimental psychology methods might provide novel insights relevant to addressing solutions to daily life problems when dealing with numbers and money quantities.

## Electronic supplementary material

Below is the link to the electronic supplementary material.

Supplementary Material 1

## Data Availability

The dataset and the reproduction codes are available at the repository: https://osf.io/8h7jr/.

## Footnotes

1 This approach is not straightforward, because money also has intrinsic value for the saving of wealth that it permits. We abstract away from this property of money.

2 We did not measure income on a continuous scale because, in our experience, this leads to substantial under-reporting of income or outright refusal to answer the question. The attrition rate can sometimes reach one quarter of the observations. For this reason, we preferred to measure income in levels. The 11 income brackets we proposed divided the income distribution for the Italian population in roughly evenly sized quartiles, as per ISTAT (the Italian National Institute of Statistics) reports. Our approach led to an attrition of 9.2%.
