# Supplementary material for: Money counts: effects of monetary vs. purely numerical values on the mental representation of quantities
Source: Psychol Res. 2025 Apr 11;89(2):85. doi: 10.1007/s00426-025-02118-z (PMC11991989; doi:10.1007/s00426-025-02118-z)
Supplement: Supplementary file 1 — Supplementary Material 1 [file 426_2025_2118_MOESM1_ESM.docx]

## Supplementary Information

### Supplementary Tables:

#### Table SI1: OLS regression analysis of the Test task including all covariates

|  | (1) | | (2) | (3) | (4) | (5) | (6) | (7) | (8) |  |
| --- | --- | --- | --- | --- | --- | --- | --- | --- | --- | --- |
| DEPENDENT VARIABLE | | R-squared linear fit: test | | | | | | | | |
| Cond_Number_fuzzy | -0.119*** | | -0.118*** | -0.148*** | -0.116*** | -0.117*** |  | -0.105 |  |  |
|  | (0.0246) | | (0.0245) | (0.0543) | (0.0239) | (0.0244) |  | (0.0985) |  |  |
| Cond _Money_fix | -0.0260 | | -0.0249 | -0.0434 | -0.0179 | -0.0185 |  | 0.118* |  |  |
|  | (0.0251) | | (0.0250) | (0.0485) | (0.0252) | (0.0256) |  | (0.0613) |  |  |
| Cond _Money_fuzzy | -0.215*** | | -0.216*** | -0.211*** | -0.216*** | -0.216*** |  | -0.194 |  |  |
|  | (0.0242) | | (0.0236) | (0.0402) | (0.0227) | (0.0230) |  | (0.119) |  |  |
| Income_med_low | 0.00118 | |  |  | -0.00527 | -0.00428 | -0.0116 | -0.00735 | -0.0130 |  |
|  | (0.0384) | |  |  | (0.0367) | (0.0371) | (0.0424) | (0.0352) | (0.0417) |  |
| Income_med_high | 0.0655** | |  |  | 0.0469* | 0.0469* | 0.0551* | 0.0491* | 0.0564* |  |
|  | (0.0299) | |  |  | (0.0274) | (0.0276) | (0.0305) | (0.0276) | (0.0301) |  |
| Income_high | 0.0191 | |  |  | -0.00234 | -0.00256 | 0.00246 | -0.00132 | 0.00300 |  |
|  | (0.0326) | |  |  | (0.0318) | (0.0321) | (0.0355) | (0.0312) | (0.0349) |  |
| Income_medium_&_high |  | | 0.0431* | 0.0271 |  |  |  |  |  |  |
|  |  | | (0.0239) | (0.0286) |  |  |  |  |  |  |
| Income_X_Number_fzy |  | |  | 0.0453 |  |  |  |  |  |  |
|  |  | |  | (0.0617) |  |  |  |  |  |  |
| Income_X_Money_fix |  | |  | 0.0273 |  |  |  |  |  |  |
|  |  | |  | (0.0532) |  |  |  |  |  |  |
| Income_X_Money_fzy |  | |  | -0.0125 |  |  |  |  |  |  |
|  |  | |  | (0.0499) |  |  |  |  |  |  |
| Shopping_scale |  | |  |  | 0.0249*** | 0.0217*** | 0.0218** | 0.0257*** | 0.0197* |  |
|  |  | |  |  | (0.00896) | (0.00832) | (0.0103) | (0.00895) | (0.0102) |  |
| Math_test_score_perc |  | |  |  | 0.0863** | 0.0857** | 0.0726 | 0.155*** | 0.147** |  |
|  |  | |  |  | (0.0414) | (0.0421) | (0.0501) | (0.0561) | (0.0673) |  |
| Shopping_Scale_X_Number_fzy |  | |  |  |  | 0.00786 |  |  |  |  |
|  |  | |  |  |  | (0.0168) |  |  |  |  |
| Shopping_Scale _X_Money_fix |  | |  |  |  | 0.00984 |  |  |  |  |
|  |  | |  |  |  | (0.0215) |  |  |  |  |
| Shopping_Scale _X_Money_fzy |  | |  |  |  | -0.00685 |  |  |  |  |
|  |  | |  |  |  | (0.0279) |  |  |  |  |
| Cond_Money |  | |  |  |  |  | -0.0488** |  | 0.0575 |  |
|  |  | |  |  |  |  | (0.0227) |  | (0.0845) |  |
| Shopping_Scale _X_Money |  | |  |  |  |  | -0.00743 |  |  |  |
|  |  | |  |  |  |  | (0.0205) |  |  |  |
| Maths _X_Number_fzy |  | |  |  |  |  |  | -0.0130 |  |  |
|  |  | |  |  |  |  |  | (0.111) |  |  |
| Maths _X_Money_fix |  | |  |  |  |  |  | -0.159** |  |  |
|  |  | |  |  |  |  |  | (0.0691) |  |  |
| Maths _X_Money_fzy |  | |  |  |  |  |  | -0.0238 |  |  |
|  |  | |  |  |  |  |  | (0.131) |  |  |
| Math_ab_X_Money |  | |  |  |  |  |  |  | -0.123 |  |
|  |  | |  |  |  |  |  |  | (0.0935) |  |
| Occupation_high_status | 0.0210 | | 0.0228 | 0.0223 | 0.0173 | 0.0158 | 0.00146 | 0.0130 | -0.00189 |  |
|  | (0.0434) | | (0.0410) | (0.0415) | (0.0450) | (0.0452) | (0.0533) | (0.0451) | (0.0530) |  |
| Occupation_medium_low_status | 0.0208 | | 0.0259 | 0.0253 | 0.0308 | 0.0290 | 0.0206 | 0.0307 | 0.0211 |  |
|  | (0.0396) | | (0.0381) | (0.0393) | (0.0412) | (0.0411) | (0.0514) | (0.0411) | (0.0514) |  |
| Occ_education | 0.0240 | | 0.0153 | 0.0154 | 0.0324 | 0.0304 | 0.0181 | 0.0380 | 0.0244 |  |
|  | (0.0311) | | (0.0283) | (0.0282) | (0.0303) | (0.0303) | (0.0399) | (0.0308) | (0.0393) |  |
| Occupation_finance | 0.0177 | | 0.0111 | 0.0104 | 0.0106 | 0.00899 | 0.0147 | 0.00705 | 0.0115 |  |
|  | (0.0199) | | (0.0193) | (0.0191) | (0.0200) | (0.0200) | (0.0243) | (0.0203) | (0.0245) |  |
| Education_master | -0.0192 | | -0.0214 | -0.0201 | -0.0266 | -0.0266 | -0.0278 | -0.0273 | -0.0290 |  |
|  | (0.0189) | | (0.0193) | (0.0195) | (0.0177) | (0.0181) | (0.0221) | (0.0172) | (0.0218) |  |
| Age | -0.00593 | | -0.00721 | -0.00822 | -0.00721 | -0.00720 | 0.00215 | -0.00737 | 0.00157 |  |
|  | (0.00642) | | (0.00625) | (0.00661) | (0.00648) | (0.00651) | (0.00749) | (0.00643) | (0.00752) |  |
| Age Squared | 7.78e-05 | | 9.10e-05 | 0.000102 | 9.46e-05 | 9.48e-05 | -9.69e-06 | 9.84e-05 | -2.30e-06 |  |
|  | (6.75e-05) | | (6.61e-05) | (6.98e-05) | (6.85e-05) | (6.88e-05) | (7.73e-05) | (6.78e-05) | (7.80e-05) |  |
| Gender | -0.0218 | | -0.0163 | -0.0160 | -0.00951 | -0.00841 | 0.00310 | -0.0132 | 0.00310 |  |
|  | (0.0211) | | (0.0217) | (0.0214) | (0.0207) | (0.0216) | (0.0237) | (0.0211) | (0.0234) |  |
| Birth_South | 0.0175 | | 0.0177 | 0.0137 | 0.0149 | 0.0138 | -0.00946 | 0.0140 | -0.0117 |  |
|  | (0.0150) | | (0.0152) | (0.0156) | (0.0147) | (0.0151) | (0.0170) | (0.0150) | (0.0171) |  |
| Married | -0.0184 | | -0.0212 | -0.0223 | -0.0298 | -0.0297 | -0.0113 | -0.0346 | -0.0174 |  |
|  | (0.0223) | | (0.0221) | (0.0226) | (0.0218) | (0.0219) | (0.0230) | (0.0215) | (0.0230) |  |
| Constant | 1.041*** | | 1.068*** | 1.102*** | 1.003*** | 1.006*** | 0.757*** | 0.949*** | 0.708*** |  |
|  | (0.141) | | (0.137) | (0.142) | (0.148) | (0.150) | (0.178) | (0.144) | (0.170) |  |
| Money conditions (aggregate) | -0.12*** | | -0.12*** | -0.11 | -0.12*** | -0.11*** |  | 0.029 |  |  |
|  | (0.040) | | (0.040) | (0.072) | (0.039) | (0.039) |  | (0.152) |  |  |
| Observations | 206 | | 206 | 206 | 206 | 206 | 206 | 206 | 206 |  |
| R-squared adj. | 0.258 | | 0.252 | 0.245 | 0.286 | 0.276 | 0.0267 | 0.292 | 0.0379 |  |

**Notes**: The dependent variable is the R^2^ of the linear interpolation of individual observations in the *Test* phase of the QPT. *Cond _Number_fuzzy, Cond _Money_fix, and Cond _Money_fuzzy* are dummy variables identifying three of the four conditions (*Treat_Number_fix* being the omitted category). *Income_med_low, Income_med_high, and Income_high* identify different income categories, as per answers to Question 6 in the questionnaire (see Supplementary Information, Section A). In particular, *Income_med_low* identify categories 4-6*, Income_med_high* identify categories 7-9, and *Income_high* identify categories 10 and 11*. Income_medium_&_high* identify income categories 7-11. *Income_X_Tr.* are interaction effects between the Income variable and the three conditions used in the analysis*. Shopping_scale* is the summative scale of answers to Questions 18, 19, and 21, 22 of the Questionnaire (Section C). *Math_test_score_perc* is the score in the 9-item test of computational abilities (see Questions 8-16 in Supplementary Information, Section B). *Shopping_Scale_X_Cond* is the interaction between *Shopping_scale* and three Condition dummies*. Maths_X_Cond.* is the interaction between *Math_test_score_perc* and three Condition dummies*. Cond_Money* is a dummy variable identifying the two Money Conditions. *Shopping_Scale__X_Money* is an interaction term between *Shopping_scale* and *Cond_Money*. Likewise, *Maths__X_Money* is an interaction term between *Math_test_score_perc* and *Cond_Money*. *Occupation_high_status* identify those occupations that lie at the top of the social status scale according to the Ganzeboom and Treiman (1996) classification (see Question 5 of the Questionnaire in the Supplementary Information, Section A). Those are, in particular, professionals, entrepreneurs, CEOs, engineers, university professors, and freelancers. *Occupation_medium_low_status* is a dummy variable identifying occupations at the medium level (e.g. private or public employees, public workers, doctoral students, teachers, researchers) and the bottom level (call centre workers, waiters, clerks) of the scale. The residual category comprises people from the workforce (e.g. students, househusbands/housewives) or unemployed. *Occ_education* is a dummy variable that identifies participants active in the educational sector, such as professors, teachers, or students. *Occupation_finance* is a dummy variable that identifies participants who are occupied in the financial sector. *Education_master* is a dummy variable identifying participants having attained at least a Master degree, as per Question 4 of the Questionnaire (see Supplementary Information, Section A). *Age* and *Age Squared* are derived from answers to Question 2 (see Supplementary Information, Section A). *Gender* identifies males. *Birth_South* identifies participants as being born in Southern Italian regions. Finally, *married* is a dummy variable that identifies married people, as per answers to Question 3 in the questionnaire. An OLS model has been fitted. Reported in parenthesis are Huber-White standard errors robust to heteroschedasticity.

#### Table SI2: Regression model with pooled Test and Retest data

| DEPENDENT VARIABLE | R-squared linear fit: test | | | | | | | | |
| --- | --- | --- | --- | --- | --- | --- | --- | --- | --- |
|  | (1) | (2) | (3) | (4) | (5) | (6) | (7) | (8) | (9) |
| Cond_Number_fuzzy | -0.119*** | -0.118*** | -0.139*** | -0.118*** | -0.118*** | -0.119*** |  | -0.0103 |  |
|  | (0.0211) | (0.0214) | (0.0426) | (0.0210) | (0.0209) | (0.0213) |  | (0.0501) |  |
| Cond _Money_fix | -0.0120 | -0.0113 | -0.0354 | -0.00755 | -0.00729 | -0.00819 |  | 0.0488 |  |
|  | (0.0159) | (0.0157) | (0.0360) | (0.0155) | (0.0156) | (0.0156) |  | (0.0585) |  |
| Cond _Money_fuzzy | -0.210*** | -0.210*** | -0.183*** | -0.211*** | -0.210*** | -0.212*** |  | -0.149** |  |
|  | (0.0193) | (0.0191) | (0.0249) | (0.0186) | (0.0187) | (0.0186) |  | (0.0658) |  |
| Income_med_low | 0.0144 |  |  | 0.0110 | 0.0106 | 0.0120 | 0.00401 | 0.0148 | 0.00419 |
|  | (0.0292) |  |  | (0.0281) | (0.0281) | (0.0284) | (0.0331) | (0.0277) | (0.0331) |
| Income_med_high | 0.0317 |  |  | 0.0210 | 0.0216 | 0.0210 | 0.0281 | 0.0236 | 0.0281 |
|  | (0.0220) |  |  | (0.0203) | (0.0203) | (0.0205) | (0.0252) | (0.0204) | (0.0251) |
| Income_high | 0.0252 |  |  | 0.0125 | 0.0129 | 0.0123 | 0.0184 | 0.0141 | 0.0180 |
|  | (0.0234) |  |  | (0.0231) | (0.0232) | (0.0231) | (0.0272) | (0.0231) | (0.0273) |
| Income_medium_&_high |  | 0.0216 | 0.0162 |  |  |  |  |  |  |
|  |  | (0.0187) | (0.0173) |  |  |  |  |  |  |
| Income_X_Number_fzy |  |  | 0.0304 |  |  |  |  |  |  |
|  |  |  | (0.0489) |  |  |  |  |  |  |
| Income_X_Money_fix |  |  | 0.0354 |  |  |  |  |  |  |
|  |  |  | (0.0391) |  |  |  |  |  |  |
| Income_X_Money_fzy |  |  | -0.0518 |  |  |  |  |  |  |
|  |  |  | (0.0366) |  |  |  |  |  |  |
| Shopping_scale |  |  |  | 0.0145* | 0.0209** | 0.0105* | 0.0119 | 0.0147* | 0.00986 |
|  |  |  |  | (0.00774) | (0.00851) | (0.00605) | (0.0105) | (0.00780) | (0.0100) |
| Math_test_score_perc |  |  |  | 0.0483 | 0.0856** | 0.0481 | 0.0304 | 0.115*** | 0.0383 |
|  |  |  |  | (0.0300) | (0.0396) | (0.0303) | (0.0363) | (0.0307) | (0.0430) |
| shopping_scale_X_test |  |  |  |  | -0.0132 |  |  |  |  |
|  |  |  |  |  | (0.0108) |  |  |  |  |
| math_test_score_perc_X_test |  |  |  |  | -0.0735 |  |  |  |  |
|  |  |  |  |  | (0.0506) |  |  |  |  |
| Shopping_Scale_X_Number_fzy |  |  |  |  |  | 0.00961 |  |  |  |
|  |  |  |  |  |  | (0.0164) |  |  |  |
| Shopping_Scale _X_Money_fix |  |  |  |  |  | 0.0105 |  |  |  |
|  |  |  |  |  |  | (0.0137) |  |  |  |
| Shopping_Scale _X_Money_fzy |  |  |  |  |  | -0.00574 |  |  |  |
|  |  |  |  |  |  | (0.0256) |  |  |  |
| Cond_Money |  |  |  |  |  |  | -0.0382* |  | -0.0269 |
|  |  |  |  |  |  |  | (0.0208) |  | (0.0597) |
| Shopping_Scale _X_Money |  |  |  |  |  |  | -0.00741 |  |  |
|  |  |  |  |  |  |  | (0.0194) |  |  |
| Maths _X_Number_fzy |  |  |  |  |  |  |  | -0.125* |  |
|  |  |  |  |  |  |  |  | (0.0643) |  |
| Maths _X_Money_fix |  |  |  |  |  |  |  | -0.0652 |  |
|  |  |  |  |  |  |  |  | (0.0620) |  |
| Maths _X_Money_fzy |  |  |  |  |  |  |  | -0.0728 |  |
|  |  |  |  |  |  |  |  | (0.0757) |  |
| Math_ab_X_Money |  |  |  |  |  |  |  |  | -0.0132 |
|  |  |  |  |  |  |  |  |  |  |
| Occupation_high_status | 0.0597 | 0.0627 | 0.0613 | 0.0584 | 0.0577 | 0.0568 | 0.0446 | 0.0572 | 0.0451 |
|  | (0.0399) | (0.0401) | (0.0400) | (0.0402) | (0.0401) | (0.0403) | (0.0481) | (0.0400) | (0.0482) |
| Occupation_medium_low_status | 0.0621* | 0.0656* | 0.0642* | 0.0690* | 0.0675* | 0.0670* | 0.0621 | 0.0669* | 0.0630 |
|  | (0.0365) | (0.0372) | (0.0379) | (0.0373) | (0.0372) | (0.0370) | (0.0453) | (0.0370) | (0.0456) |
| Occ_education | 0.0279 | 0.0269 | 0.0267 | 0.0328 | 0.0330 | 0.0308 | 0.0178 | 0.0392* | 0.0189 |
|  | (0.0213) | (0.0207) | (0.0211) | (0.0208) | (0.0209) | (0.0211) | (0.0281) | (0.0213) | (0.0275) |
| Occupation_finance | 0.00821 | 0.00746 | 0.00771 | 0.00391 | 0.00454 | 0.00222 | 0.00640 | 0.00431 | 0.00622 |
|  | (0.0163) | (0.0158) | (0.0156) | (0.0166) | (0.0166) | (0.0167) | (0.0215) | (0.0168) | (0.0217) |
| Education_master | -0.00512 | -0.00518 | -0.00374 | -0.00986 | -0.00933 | -0.00975 | -0.00894 | -0.00876 | -0.00900 |
|  | (0.0153) | (0.0155) | (0.0152) | (0.0154) | (0.0154) | (0.0155) | (0.0200) | (0.0151) | (0.0199) |
| Age | -0.00547 | -0.00551 | -0.00766 | -0.00630 | -0.00611 | -0.00628 | 0.00336 | -0.00684 | 0.00314 |
|  | (0.00552) | (0.00534) | (0.00559) | (0.00569) | (0.00570) | (0.00563) | (0.00689) | (0.00571) | (0.00691) |
| Age Squared | 6.14e-05 | 6.23e-05 | 8.66e-05 | 7.23e-05 | 6.98e-05 | 7.24e-05 | -3.50e-05 | 7.80e-05 | -3.26e-05 |
|  | (5.80e-05) | (5.63e-05) | (5.91e-05) | (6.05e-05) | (6.07e-05) | (5.99e-05) | (7.19e-05) | (6.07e-05) | (7.25e-05) |
| Gender | -0.0228 | -0.0228 | -0.0220 | -0.0155 | -0.0160 | -0.0144 | -0.00208 | -0.0129 | -0.00208 |
|  | (0.0176) | (0.0179) | (0.0174) | (0.0173) | (0.0173) | (0.0176) | (0.0210) | (0.0176) | (0.0210) |
| Birth_South | 0.00946 | 0.00990 | 0.00552 | 0.00809 | 0.00803 | 0.00692 | -0.0178 | 0.00849 | -0.0184 |
|  | (0.0130) | (0.0130) | (0.0128) | (0.0128) | (0.0128) | (0.0134) | (0.0152) | (0.0129) | (0.0153) |
| Married | -0.0251 | -0.0250 | -0.0263 | -0.0320* | -0.0312* | -0.0319* | -0.0144 | -0.0311* | -0.0158 |
|  | - | (0.0173) | (0.0177) | (0.0175) | (0.0174) | (0.0175) | (0.0206) | (0.0169) | (0.0205) |
| Retest | 0.0254** | 0.0254** | 0.0252** | 0.0253** | 0.0887* | 0.0253** | 0.0264** | 0.0254** | 0.0264** |
|  | (0.0103) | (0.0103) | (0.0103) | (0.0103) | (0.0459) | (0.0103) | (0.0103) | (0.0103) | (0.0103) |
| Constant | 1.010*** | 1.014*** | 1.064*** | 0.991*** | 0.955*** | 0.993*** | 0.736*** | 0.942*** | 0.734*** |
|  | (0.125) | (0.122) | (0.124) | (0.130) | (0.133) | (0.129) | (0.157) | (0.127) | (0.156) |
| Observations | 415 | 415 | 415 | 415 | 415 | 415 | 415 | 415 | 415 |
| R-squared adj. | 0.285 | 0.284 | 0.295 | 0.296 | 0.302 | 0.297 | 0.0554 | 0.300 | 0.0554 |

**Note**: An OLS panel estimator has been fitted to data merging Test and Retest responses. The variable “Retest Decision” identifies responses from the Restest. Standard errors have been clustered at the individual level to correct for the auto-correlations of standard errors. *Shopping_scale_X_retest* and *Math_test_score_perc_X_retest* are interaction terms of *Shopping_scale* and *Math_test_score_perc* with the retest response. See Note to Table 2 in the main paper to describe the other variables.

#### Table SI3: Regression model with pooled Test and Retest data - Interaction effects

| DEPENDENT VARIABLE | R-squared linear fit: test & Retest | | | | | |
| --- | --- | --- | --- | --- | --- | --- |
|  | (1) | (2) | (3) | (4) | (5) | (6) |
| Cond_Number_fuzzy | -0.118*** | -0.118*** | -0.119*** | -0.125*** | -0.118*** | -0.118*** |
|  | (0.0210) | (0.0210) | (0.0231) | (0.0230) | (0.0210) | (0.0209) |
| Cond _Money_fix | -0.00753 | -0.0154 | -0.00756 | -0.0141 | -0.00752 | -0.00729 |
|  | (0.0155) | (0.0230) | (0.0155) | (0.0200) | (0.0155) | (0.0155) |
| Cond _Money_fuzzy_X_Retest | -0.209*** | -0.211*** | -0.211*** | -0.218*** | -0.211*** | -0.210*** |
|  | (0.0221) | (0.0187) | (0.0187) | (0.0205) | (0.0186) | (0.0187) |
| Cond_Number_fuzzy_X_Retest | -0.00532 |  |  |  |  |  |
|  | (0.0322) |  |  |  |  |  |
| Cond _Money_fix_X_Retest |  | 0.0157 |  |  |  |  |
|  |  | (0.0228) |  |  |  |  |
| Cond _Number_fuzzy_X_Retest |  |  | 0.00145 |  |  |  |
|  |  |  | (0.0240) |  |  |  |
| Cond _Number_fix_X_Retest |  |  |  | -0.0130 |  |  |
|  |  |  |  | (0.0165) |  |  |
| Shopping_scale | 0.0145* | 0.0145* | 0.0145* | 0.0145* | 0.0225*** | 0.0143* |
|  | (0.00775) | (0.00775) | (0.00774) | (0.00775) | (0.00853) | (0.00771) |
| shopping_scale_X_Retest |  |  |  |  | -0.0160 |  |
|  |  |  |  |  | (0.0108) |  |
| Math_test_score_perc | 0.0484 | 0.0483 | 0.0484 | 0.0481 | 0.0488 | 0.0875** |
|  | (0.0299) | (0.0300) | (0.0301) | (0.0301) | (0.0300) | (0.0396) |
| math_test_score_perc_X_Retest |  |  |  |  |  | -0.0782 |
|  |  |  |  |  |  | (0.0502) |
| Income_med_low | 0.0109 | 0.0110 | 0.0109 | 0.0109 | 0.0110 | 0.0105 |
|  | (0.0281) | (0.0281) | (0.0281) | (0.0281) | (0.0281) | (0.0281) |
| Income_med_high | 0.0210 | 0.0210 | 0.0210 | 0.0210 | 0.0210 | 0.0217 |
|  | (0.0203) | (0.0203) | (0.0203) | (0.0203) | (0.0203) | (0.0203) |
| Income_high | 0.0125 | 0.0124 | 0.0125 | 0.0125 | 0.0125 | 0.0129 |
|  | (0.0231) | (0.0231) | (0.0231) | (0.0231) | (0.0231) | (0.0231) |
| Occupation_high_status | 0.0583 | 0.0583 | 0.0583 | 0.0585 | 0.0585 | 0.0576 |
|  | (0.0403) | (0.0402) | (0.0402) | (0.0403) | (0.0403) | (0.0401) |
| Occupation_medium_low_status | 0.0689* | 0.0689* | 0.0689* | 0.0691* | 0.0692* | 0.0672* |
|  | (0.0373) | (0.0373) | (0.0372) | (0.0373) | (0.0374) | (0.0371) |
| Occ_education | 0.0328 | 0.0328 | 0.0328 | 0.0328 | 0.0328 | 0.0330 |
|  | (0.0208) | (0.0208) | (0.0208) | (0.0208) | (0.0208) | (0.0208) |
| Occupation_finance | 0.00390 | 0.00390 | 0.00396 | 0.00390 | 0.00387 | 0.00458 |
|  | (0.0165) | (0.0166) | (0.0166) | (0.0166) | (0.0166) | (0.0165) |
| Education_master | -0.00988 | -0.00995 | -0.00992 | -0.00986 | -0.00996 | -0.00917 |
|  | (0.0154) | (0.0154) | (0.0154) | (0.0154) | (0.0154) | (0.0154) |
| Age | -0.00631 | -0.00632 | -0.00630 | -0.00630 | -0.00633 | -0.00607 |
|  | (0.00570) | (0.00569) | (0.00570) | (0.00570) | (0.00569) | (0.00570) |
| Age Squared | 7.24e-05 | 7.25e-05 | 7.23e-05 | 7.23e-05 | 7.27e-05 | 6.94e-05 |
|  | (6.06e-05) | (6.06e-05) | (6.06e-05) | (6.06e-05) | (6.06e-05) | (6.06e-05) |
| Gender | -0.0155 | -0.0155 | -0.0155 | -0.0155 | -0.0154 | -0.0162 |
|  | (0.0173) | (0.0173) | (0.0173) | (0.0173) | (0.0173) | (0.0173) |
| Birth_South | 0.00811 | 0.00812 | 0.00809 | 0.00804 | 0.00807 | 0.00804 |
|  | (0.0129) | (0.0129) | (0.0128) | (0.0129) | (0.0129) | (0.0128) |
| Married | -0.0320* | -0.0321* | -0.0320* | -0.0320* | -0.0321* | -0.0312* |
|  | (0.0174) | (0.0175) | (0.0175) | (0.0175) | (0.0175) | (0.0174) |
| Retest | 0.0263** | 0.0213* | 0.0248** | 0.0285** | 0.0258** | 0.0924** |
|  | (0.0103) | (0.0122) | (0.0118) | (0.0133) | (0.0104) | (0.0458) |
| Constant | 0.991*** | 0.994*** | 0.992*** | 0.996*** | 0.991*** | 0.953*** |
|  | (0.130) | (0.130) | (0.130) | (0.130) | (0.130) | (0.133) |
| Observations | 415 | 415 | 415 | 415 | 415 | 415 |
| Number of id | 211 | 211 | 211 | 211 | 211 | 211 |
| R-squared adj-Within | 0.0384 | 0.0389 | 0.0383 | 0.0404 | 0.0471 | 0.0559 |
| R-squared adj-Between | 0.353 | 0.354 | 0.353 | 0.352 | 0.352 | 0.355 |
| R-squared adj-Overall | 0.296 | 0.297 | 0.296 | 0.296 | 0.298 | 0.301 |

#### Table SI4: Regression models derived from LASSO model selection techniques and basic specification with 11 levels for income

| **DEPENDENT VARIABLE** | **R-squared linear fit: test** | | |
| --- | --- | --- | --- |
|  | (1) | (2) | (3) |
| Cond_Number_fuzzy | -0.111*** | -0.116*** | -0.117*** |
|  | (0.0244) | (0.0247) | (0.0229) |
| Cond _Money_fix |  |  | -0.0146 |
|  |  |  | (0.0265) |
| Cond _Money_fuzzy | -0.218*** | -0.221*** | -0.218*** |
|  | (0.0238) | (0.0258) | (0.0228) |
| Shopping_scale | 0.0199** | 0.0241*** | 0.0252*** |
|  | (0.00804) | (0.00779) | (0.00897) |
| Math_test_score_perc | 0.124*** | 0.120*** | 0.0818* |
|  | (0.0409) | (0.0437) | (0.0428) |
| Income_2 |  |  | -0.0910 |
|  |  |  | (0.0620) |
| Income_3 |  |  | -0.100** |
|  |  |  | (0.0443) |
| Income_4 |  |  | -0.0680* |
|  |  |  | (0.0389) |
| Income_5 |  |  | -0.0871 |
|  |  |  | (0.0691) |
| Income_6 |  |  | -0.00556 |
|  |  |  | (0.0336) |
| Income_7 |  |  | -0.0340 |
|  |  |  | (0.0336) |
| Income_8 |  |  | -0.0761* |
|  |  |  | (0.0407) |
| Income_9 |  |  | -0.0585 |
|  |  |  | (0.0391) |
| Income_10 |  |  | -0.110 |
|  |  |  | (0.0840) |
| Income_11 |  |  | -0.0690 |
|  |  |  | (0.0439) |
| Occupation_high_status |  |  | 0.0217 |
|  |  |  | (0.0435) |
| Occupation_medium_low_status |  |  | 0.0301 |
|  |  |  | (0.0398) |
| Occ_education |  |  | 0.0411 |
|  |  |  | (0.0317) |
| Occupation_finance |  |  | 0.0144 |
|  |  |  | (0.0207) |
| Education_master | -0.0265 | -0.0238 | -0.0240 |
|  | (0.0175) | (0.0183) | (0.0166) |
| Age | -0.0107* | -0.0100 | -0.00866 |
|  | (0.00580) | (0.00639) | (0.00658) |
| Age Squared | 0.000129** | 0.000123* | 0.000108 |
|  | (6.31e-05) | (6.84e-05) | (6.89e-05) |
| Gender |  |  | -0.00487 |
|  |  |  | (0.0202) |
| Birth_South |  | 0.00147 | 0.00793 |
|  |  | (0.0196) | (0.0147) |
| Married |  | -0.0189 | -0.0247 |
|  |  | (0.0212) | (0.0217) |
| Income_med_high | 0.0437** | 0.0489*** |  |
|  | (0.0175) | (0.0178) |  |
| Constant | 1.061*** | 1.053*** | 1.104*** |
|  | (0.128) | (0.143) | (0.145) |
| Observations | 231 | 221 | 206 |
| R-squared | 0.332 | 0.331 | 0.368 |

**Note:** The first two columns report models selected with Lasso Covariance and Selective techniques, respectively. The third column reports the basic model from TableSI2, column 4, modified by replacing the three-level income variable with the 11-level income variable included in the questionnaire.

### Supplementary Analysis on pooled Test and Retest data

Following the suggestions of a Reviewer, we report the analysis collapsing test and retest conditions.

Group analyses on median PAE (complementary to section 3.2):

- Money-Fuzzy: R2 lin-fit= 0.75; R2 log-fit=0.99; comparison of residuals: t(6)=3.2; p<0.01;
- Money-Fixed: R2 lin-fit= 0.998; R2 log-fit=0.75; comparison of residuals: t(6)=-4.03; p<0.01;
- Number-Fuzzy: R2 lin-fit= 0.98; R2 log-fit=0.84; comparison of residuals: t(6)=-2.95; p<0.05;
- Number-Fixed: R2 lin-fit= 0.999; R2 log-fit=0.74; comparison of residuals: t(6)=-4.1; p<0.01.

Comparison of Money vs. Number PAE collapsing test and retest (complementary to section 3.3):

- Fuzzy 5: z=-1.96; p=.0498;
- Fuzzy 10: z=-4.267; p<.0001;
- Fuzzy 20: z=-4.754; p<.0001;
- Fuzzy 50: z=-4.217; p<.0001;
- Fuzzy 100: z=-4.897; p<.0001;
- Fuzzy 200: z=-4.7; p<.0001;
- Fuzzy 500: z=0.6; p=.5;
- Fixed 5: z=-2.934; p<.001;
- Fixed 10: z=-3.955; p<.0001;
- Fixed 20: z=-3.104; p<.01;
- Fixed 50: z=-3.02; p<.01;
- Fixed 100: z=-2.294; p<.05;
- Fixed 200: z=-1.776; p=.08;
- Fixed 500: z=0.316; p=.8.

### Test Materials:

### Section A:

#### Questionnaire (extract)

##### Demographics

1. Please indicate your gender: 🞏_1_ Male 🞏_2_Female
2. Please indicate your year of birth: ______________
3. Please indicate your civil status:

🞏_1_ Single 🞏_2_Married or cohabiting 🞏_3_ Separated or divorced 🞏_4_ Widow/widower

1. Which is the highest level of education that you achieved?

| Primary School | Secondary School-inferior | Secondary School-superior | University Bachelor degree – first level | University Bachelor degree – second level | Master | Doctoral studies |
| --- | --- | --- | --- | --- | --- | --- |
| 🞏_1_ | 🞏_2_ | 🞏_3_ | 🞏_4_ | 🞏_5_ | 🞏_6_ | 🞏_7_ |

1. What is your occupation? If you are currently retired or unemployed, which was your last occupation?
2. Could you please indicate your household annual income (taking into account all your salaries, pensions and other incomes, net of taxes and other deductions)? By household, we mean all family members with whom you usually reside.

| E. 0-12,000 | 12,000-15,000 | 15,000-19,000 | 19,000-23,000 | 23,000-27,000 | 27,000-32,000 | 32,000-39,000 | 39,000-46,000 | 46,000-57,000 | 57,000- 70,000 | Above  70,000 |
| --- | --- | --- | --- | --- | --- | --- | --- | --- | --- | --- |
| 🞏_1_ | 🞏_2_ | 🞏_3_ | 🞏_4_ | 🞏_5_ | 🞏_6_ | 🞏_7_ | 🞏_8_ | 🞏_9_ | 🞏_10_ | 🞏_11_ |

1. How many people there are in your household?

##### Section B: Math ability quiz

1. Which is the greatest, 1/10 or 9/80?
2. How much is 20% of two Euros?
3. Which is the greatest,11/89 or 10/90?
4. How much is 1/5 of two Euros?
5. How much is 89% of 100 euro?
6. How much is 11% of 500 euro?
7. Imagine a six-sided die is thrown 1000 times. Over such 1000 throws, how many times can you expect an even number (2,4,6) to come out?
8. In the lottery A, the probability of winning 10 Euros is 1%. How many people can win the prize if 1000 people buy a ticket each?
9. In the lottery B, the probability of winning a car is one over 1000. Which percentage of lottery B tickets allows one to win a car?

**Section C: Spending habits and practice with financial investment**

1. How frequently do you go to the grocery store to buy food or products for daily consumption (e.g., bread, beverages, paper) for you and your family?

🞏_1_More than once a week

🞏_2_From two to four times a month

🞏_3_About once a month

🞏_4_Less than once a month

1. How much do you usually spend on these occasions?
2. How much did you spend the last time you went?
3. How often do you shop to buy durable products (e.g., clothing, mobile phones) for you or your family?

🞏_1_ About once a week

🞏_2_ About once a month

🞏_3_ From two to four times a year

🞏_4_ Less than twice a year

1. How much do you usually spend on these occasions?
2. How much did you spend last time?
3. Do you usually pay in cash?

🞏_1_ I almost always pay in cash

🞏_2_ I pay either in cash or by cheque/cash/credit card

🞏_3_ I almost always pay by cheque/cash/credit card

1. How many credit cards do you have?

🞏_1_ None

🞏_2_ One

🞏_3_ Two to four

🞏_4_ More than four

1. Are you generally available to take risks in managing your savings, or do you try to avoid risks?

🞏_1_ I am fully prepared to take risks

🞏_2_ I take risks on some occasions

🞏_3_ I try to avoid taking risks

1. How are you doing financially in this period?

🞏_1_ Well, I am living comfortably

🞏_2_ Well, I live in acceptable conditions

🞏_3_ I can barely get by

🞏_4_ I am doing badly

🞏_5_ I am doing really badly

1. What is the percentage of your savings that you keep invested in financial markets?
2. How often do you manage financial instruments (shares, bonds, futures, derivatives ...)?

🞏_1_ Never

🞏_2_ Once a year or every six month

🞏_3_ Once every 4 month

🞏_4_ Monthly

🞏_5_ Weekly

🞏_6_ Daily

1. Which is the goal of your investment?

🞏_1_ Financial gain in the long run

🞏_2_ Have constant revenues to integrate current expenses

🞏_3_ Grant me an extra income for my pension

🞏_4_ Financial gain in the short term

🞏_5_ To cover an unexpected expense

🞏_6_ Pay for the purchase of durable goods

🞏_7_ Other: ………………………………………………………………………

1. Which financial instrument do you know?

🞏_1_ Bonds outside Europe

🞏_2_ Funds

🞏_3_ European corporate bonds

🞏_4_ Futures and listed derivatives

🞏_5_ Equity funds

🞏_6_ Italian Stocks

🞏_7_ OTC derivatives

🞏_8_ Hedge Funds

🞏_9_ Foreign equities

🞏_10_ Balanced funds

🞏_11_ asset allocation

🞏_12_ Bond funds

🞏_13_ Sovereign European bonds (government bonds)

🞏_14_ Covered Warrants image ETF

🞏_15_ Other: ……………………………………………………………………….

1. Do you do gambling?

🞏_1_ Yes

🞏_2_ Sometimes

🞏_3_ Rarely

🞏_4_ Very rarely

🞏_5_ Never

1. If you gamble, what do you play?

🞏_1_ Playing cards with friends (poker, seven-thirty, etc.)

🞏_2_ Casino

🞏_3_ New Year’s national lottery

🞏_4_ Sport betting (horse racing, football, etc.)

🞏_5_ Video Poker

🞏_6_ Lotto

🞏_7_ Other national lotteries

🞏_8_ Other: ……………………………………………………………………...

1. Do you feel you are a lucky person?

Very unlucky Very lucky

| 1 | 2 | 3 | 4 | 5 | 6 | 7 | 8 | 9 | 10 |
| --- | --- | --- | --- | --- | --- | --- | --- | --- | --- |
